# Design, Synthesis, and Biological Activity of New CB2 Receptor Ligands: from Orthosteric and Allosteric Modulators to Dualsteric/Bitopic Ligands

**DOI:** 10.1021/acs.jmedchem.2c00582

**Published:** 2022-07-18

**Authors:** Francesca Gado, Rebecca Ferrisi, Beatrice Polini, Kawthar A. Mohamed, Caterina Ricardi, Elena Lucarini, Sara Carpi, Federica Domenichini, Lesley A. Stevenson, Simona Rapposelli, Giuseppe Saccomanni, Paola Nieri, Gabriella Ortore, Roger G. Pertwee, Carla Ghelardini, Lorenzo Di Cesare Mannelli, Grazia Chiellini, Robert B. Laprairie, Clementina Manera

**Affiliations:** † Department of Pharmacy, University of Pisa, Pisa 56126, Italy; ‡ Department of Pathology, University of Pisa, Pisa 56126, Italy; § College of Pharmacy and Nutrition, University of Saskatchewan, Saskatoon SK S7N 5E5, Canada; ∥ Department of Neuroscience, Psychology, Drug Research and Child Health, Section of Pharmacology and Toxicology, University of Florence, Florence 50139, Italy; ⊥ NEST, Istituto Nanoscienze-CNR and Scuola Normale Superiore, Piazza San Silvestro, Pisa 56126, Italy; # Institute of Medical Sciences, University of Aberdeen, Aberdeen AB25 2ZD, Scotland, U.K.; ¶ CISUP, Centre for Instrumentation Sharing Pisa University, Lungarno Pacinotti 43, Pisa 56126, Italy; ∇ Department of Pharmacology, College of Medicine, Dalhousie University, Halifax B3H 4R2, Nova Scotia, Canada

## Abstract

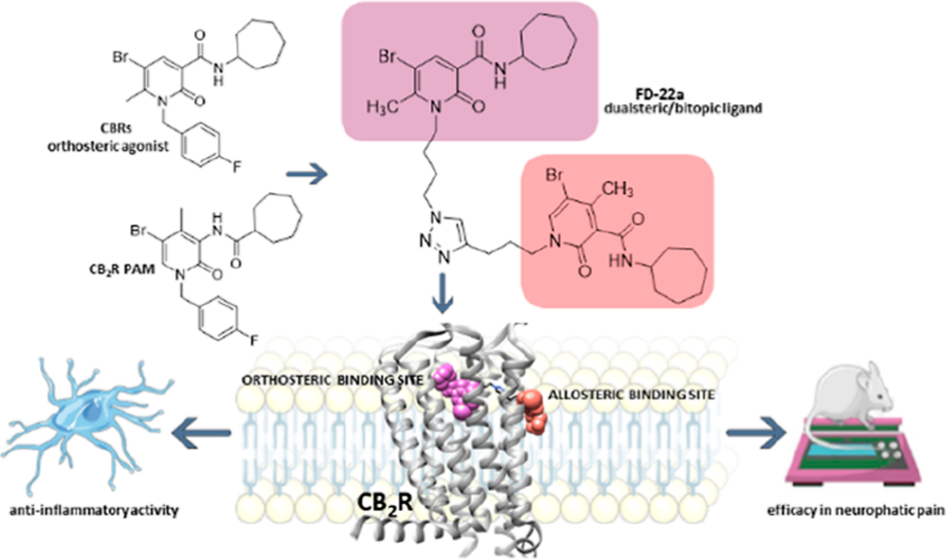

The design of dualsteric/bitopic agents as single chemical entities able to simultaneously interact with both the orthosteric and an allosteric binding site represents a novel approach in medicinal chemistry. Biased dualsteric/bitopic agents could enhance certain signaling pathways while diminishing the others that cause unwanted side effects. We have designed, synthesized, and functionally characterized the first CB2R heterobivalent bitopic ligands. In contrast to the parent orthosteric compound, our bitopic ligands selectively target CB2R versus CB1R and show a functional selectivity for the cAMP signaling pathway versus βarrestin2 recruitment. Moreover, the most promising bitopic ligand **FD-22a** displayed anti-inflammatory activity in a human microglial cell inflammatory model and antinociceptive activity *in vivo* in an experimental mouse model of neuropathic pain. Finally, computational studies clarified the binding mode of these compounds inside the CB2R, further confirming their bitopic nature.

## Introduction

G protein–coupled receptors (GPCRs) constitute the largest family of membrane receptors in the human genome^1^ and are divided into five families: rhodopsin, secretin, glutamate, adhesion, and frizzled/TAS2.^2^ GPCRs are ubiquitous cell surface proteins that respond to a wide variety of ligands contributing to multiple physiological and pathophysiological processes. As a consequence of their ubiquitous distribution, GPCRs have frequently been exploited as attractive drug targets, currently accounting for around approximately 30% of all FDA-approved drugs.^3,4^ Only around one-fifth of the total complement of GPCRs in the genome have been established as therapeutic targets, thus indicating that new potential drugs among this important family of receptors might be discovered.^5^ The biggest challenge facing medicinal chemists is the development of ligands able to selectively target a specific GPCR subtype.^6^ Indeed, structurally, all GPCRs share a characteristic architecture: seven transmembrane-spanning helical domains, which have evolved to accommodate the dual roles of extracellular ligand recognition and intracellular signal transduction.^5^ Despite this common structure, however, GPCRs present an enormous functional versatility, responding to light, ions, odorant molecules, biogenic amines, lipids, nucleotides, peptides, large proteins, and many other molecules, owing to their conformational flexibility which allows them to assume multiple conformations.^7^

Traditionally, medicinal chemistry approaches focused on the binding sites, known as orthosteric sites, usually recognized by endogenous ligands. Unfortunately, orthosteric sites are likely to be highly conserved across GPCR subtypes.^5^ The binding between GPCRs and orthosteric sites leads to conformational changes at the cytoplasmic ends of the GPCRs’ domains, providing an interaction surface for intracellular adaptor proteins, such as heterotrimeric G proteins, G protein-coupled receptor kinases (GRKs), and βarrestins, with each of them being responsible for different signaling cascades.

Some ligands may stabilize subsets of receptor conformations that favor diverse functional outcomes and induce particular signaling pathways at the expense of others. This results in a unique ligand-dependent signaling profile, a scenario which is also referred to as functional selectivity, biased agonism, or stimulus bias.^8,9^ Biased GPCR ligands have been shown to display beneficial biological responses in preclinical and clinical studies, which explains the growing interests of medicinal chemists in biased signaling.^10,11^

These promising examples for the translation of biased agonists to beneficial biological responses lead to the development of innovative approaches to engender both subtype and functional selectivity at GPCRs.^12^ With this aim, researchers have explored the effectiveness of targeting topographically distinct and less conserved binding sites, namely “allosteric” sites. GPCR allosteric sites are of interest in research because they are not subjected to the same evolutionary pressures as orthosteric sites. Therefore, targeting allosteric sites may allow for a greater subtype selectivity. However, this strategy is hampered by the lack of knowledge regarding allosteric site(s), location, and structure.^13,14^ Allosteric modulators devoid of intrinsic activity alter receptor signaling through conformational changes in the receptor, modifying the affinity and/or efficacy of an orthosteric ligand without showing intrinsic effects *per se*. One unique feature of allosteric ligands is agonist dependence, better known as probe dependence, ultimately implying that the same allosteric ligand may differentially modulate the activity of different orthosteric ligands.^15,16^ Moreover, allosteric ligands may alter the specific signal bias of orthosteric ligands or display ligand bias themselves.^16−19^ Allosteric ligands can be classified as positive (PAM), negative (NAM), or neutral (NAL) allosteric modulators depending on the type of modulation on the affinity and/or efficacy of the orthosteric ligand. Allosteric ligands have been found to present a generally reduced side effect profile compared to orthosteric ligands but often suffer from reduced efficacy.^17,20^ To overcome this problem, a new approach in medicinal chemistry is to encode both orthosteric and allosteric properties within a single therapeutic agent, a bitopic/dualsteric ligand, that bridges two topographically distinct ligand-binding domains. Bitopic/dualsteric ligands are hybrid compounds consisting of two distinct pharmacophores which are connected by a linker, allowing simultaneous binding to the orthosteric and allosteric sites of the same receptor.^21,22^ This strategy was derived from the “message–address” concept of Schwyzer, published in the 1970s,^23^ in which the message part activates the receptor, while the address part leads the ligand specifically to the receptor, or receptor-subtype of interest. A bitopic/dualsteric ligand is then developed when the “message” occupies the highly conserved orthosteric area while the “address” binds to the less conserved allosteric binding pocket.^24^ Consequently, bitopic/dualsteric compounds may present interesting advantages, including the potential for greater receptor selectivity by virtue of targeting an allosteric site, and greater affinity due to the concomitant engagement with the orthosteric site. Bitopic/dualsteric ligands may therefore prove to be particularly useful in situations where endogenous agonist tone is progressively lost, such as in neurodegenerative disorders,^25^ thanks to the co-presence of the orthosteric and the allosteric modulator. In addition, the administration of a bitopic/dualsteric compound may have additive or synergistic therapeutic effects, leading to the use of a lower dose as compared to the single dose of each parent compound. However, the development of bitopic/dualsteric compounds presents several problems related to the fact that the orthosteric and allosteric parts, as well as the linker, must be optimized and connected appropriately to obtain bitopic/dualsteric compounds with increased efficacy, affinity, and selectivity with respect to the parent compounds.^24,26,27^ The choice of the correct linker plays a crucial role. Indeed, the linker must be specially made respecting length, flexibility, and chemical properties. The linker must have the right length in order to allow the two pharmacophores to interact correctly with the respective binding site and avoid steric hindrance problems. Bitopic/dualsteric compounds may also present novel biased properties because the incorporation of two pharmacophores in one ligand can severely impact receptor flexibility and thus signaling output.^24,28,29^

In this work, we focused on the development of bitopic/dualsteric ligands for the cannabinoid receptor 2 (CB2R), a GPCR belonging, together with the cannabinoids receptor 1 (CB1R), to the endocannabinoid system (ECS). The ECS is known to play fundamental roles in neurophysiology and nociception.^30^ Taking into account that the pharmacological activation of CB2R has been recently shown to produce several neuroprotective effects without causing psychotropic adverse effects, frequently associated with the stimulation of CB1R, targeting CB2R might provide a new and safer approach to the treatment of neurodegenerative disorders and pain.^31^

**Figure 1:**
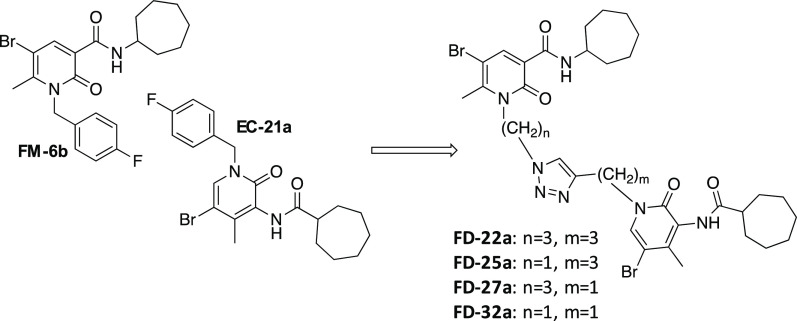
Design of compounds **FD-22a, FD-25a, FD-27a,** and **FD-32a**.

Over the past two decades, considerable efforts have been made in developing ligands for both cannabinoid receptors subtypes, leading to hundreds of synthetic cannabinoids which have displayed a wide array of biological effects, signifying broad therapeutic potentials. Nevertheless, only a limited number of ligands are clinically applicable. Our group has already developed a small library of CBR orthosteric ligands.^32−34^ Among them the 2-oxo-pyridine derivative **FM-6b** (Figure 1) was the most promising, acting as a full agonist at both CBRs with high affinity.^34^ Functional studies also revealed a significant activity of **FM-6b** on neuroinflammation, glutamate-mediated excitotoxicity and neuropathic pain.^34^ Interestingly, in mouse microglial cells exposed to lipopolysaccharide (LPS) insult, a CB2R-dependent reduction of proinflammatory interleukin secretion was also observed after treatment with **FM-6b**.^34^ Moreover, we recently reported the identification of a novel 2-oxopyridine-3-carboxamide derivative, namely **EC-21a**, as the first small synthetic CB2R positive allosteric modulator (PAM) (i.e., CB2R PAM).^35^ Indeed, as expected for an allosteric modulator, **EC-21a** elicited a marked increase in the binding of the high-affinity nonselective radioligand [^3^H]CP55, 940 to CB2R and in the ability of CP55, 940 to stimulate [^35^S]GTPγS binding to CB2R, along with the absence of effects on CB2R signaling in [^35^S]GTPγS assays carried out in the absence of a CB2R agonist.^35^Additionally, *in vivo* experiments revealed **EC-21a** efficacy in reducing neuropathic pain^35^ and increased resistance to induced seizures in CF1 wildtype mice and mice harboring the *scn1a* R1648H human epilepsy mutation.^36^

Finally, we recently provided compelling evidence that the combination of the dual orthosteric CB1R/CB2R agonist **FM-6b** with CB2R PAM **EC-21a** enhanced the ability of the orthosteric agonist **FM-6b** to modulate the release of pro- and anti-inflammatory interleukins in LPS-activated mouse BV2 microglial cells.^37^ Notably, the observed combination therapy effect was completely abolished after pretreatment with the CB2R antagonist SR144528, further confirming a CB2R-mediated effect.^37^

On the basis of these findings, we decided to synthesize a new series of potential CB2R bitopic/dualsteric ligands, namely **FD-22a, FD-25a, FD-27a,** and **FD-32a** (Figure 1), by linking the pharmacophoric portion of CB2R PAM **EC-21a** to that of the CB2R orthosteric agonist **FM-6b**. Notably, both parent compounds were modified at position N(1) on their central core structure to allow the introduction of a designed linker. Concerning to this aspect, previous structural activity relation studies indicated this position as the most suitable to chemical modifications without significantly compromising activity.^19,33−35^ Structurally, the linker consists of a disubstituted 1,2,3- triazole ring connected to two alkyl chain of variable length at position N(1) and C(4), respectively. Among nitrogen-containing heterocyclic compounds, 1,2,3-triazoles have found broad applications in drug discovery.^38^ In particular, 1,2,3-triazoles are stable toward metabolic degradation and easily form hydrogen bonding, which can increase solubility favoring the binding of biomolecular targets.^39^

Among all the newly designed compounds, *in vitro* assays indicated the derivative **FD-22a** as the most promising CB2R bitopic/dualsteric ligands. Consequently, compound **FD-22a** was further exposed to additional functional assays and *in vivo* tests.

## Results and Discussion

### Chemistry

**Scheme 1:**
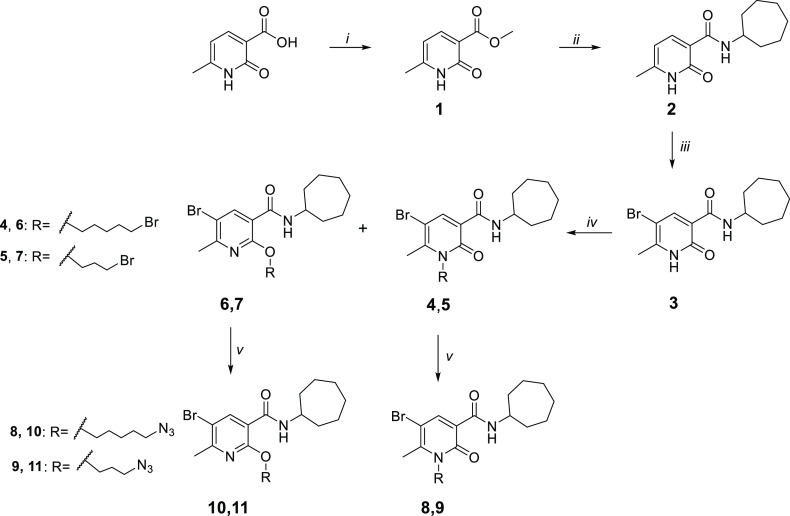
Synthetic Pathway for the Synthesis of the Azido Derivatives **8-11** Reagents and conditions: (i) MeOH, H_2_SO_4_ 96%, 90 °C, 24 h. (ii) cycloheptylamine, 100 °C, 24 h. (iii) Br_2_, CHCl_3_, rt, 12 h. (iv) (a) CsF, DMF, rt, 1 h (b) R-bromide, 50 °C, 12 h. (v) NaN_3_, DMF, 60 °C, 12 h.

**Scheme 2:**
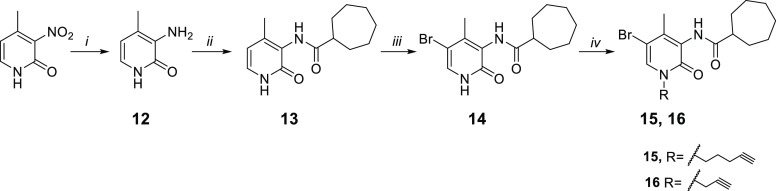
Synthetic Pathway for the Synthesis of the Alkyne Derivatives **15** and **16** Reagents and conditions: (i) Fe, NH_4_Cl, H_2_O/EtOH 1:2, 80 °C, 3 h. (ii) (a) cycloheptanecarboxylic acid, C_2_O_2_Cl_2_, DMF, rt, 0.5 h (b) NEt_3_, DCM, DMF, rt, 24 h. (iii) Br_2_, CHCl_3_, rt, 12 h. (iv) (a) CsF, DMF, rt, 1 h (b) R-bromide, 30 °C, 12 h.

**Scheme 3:**
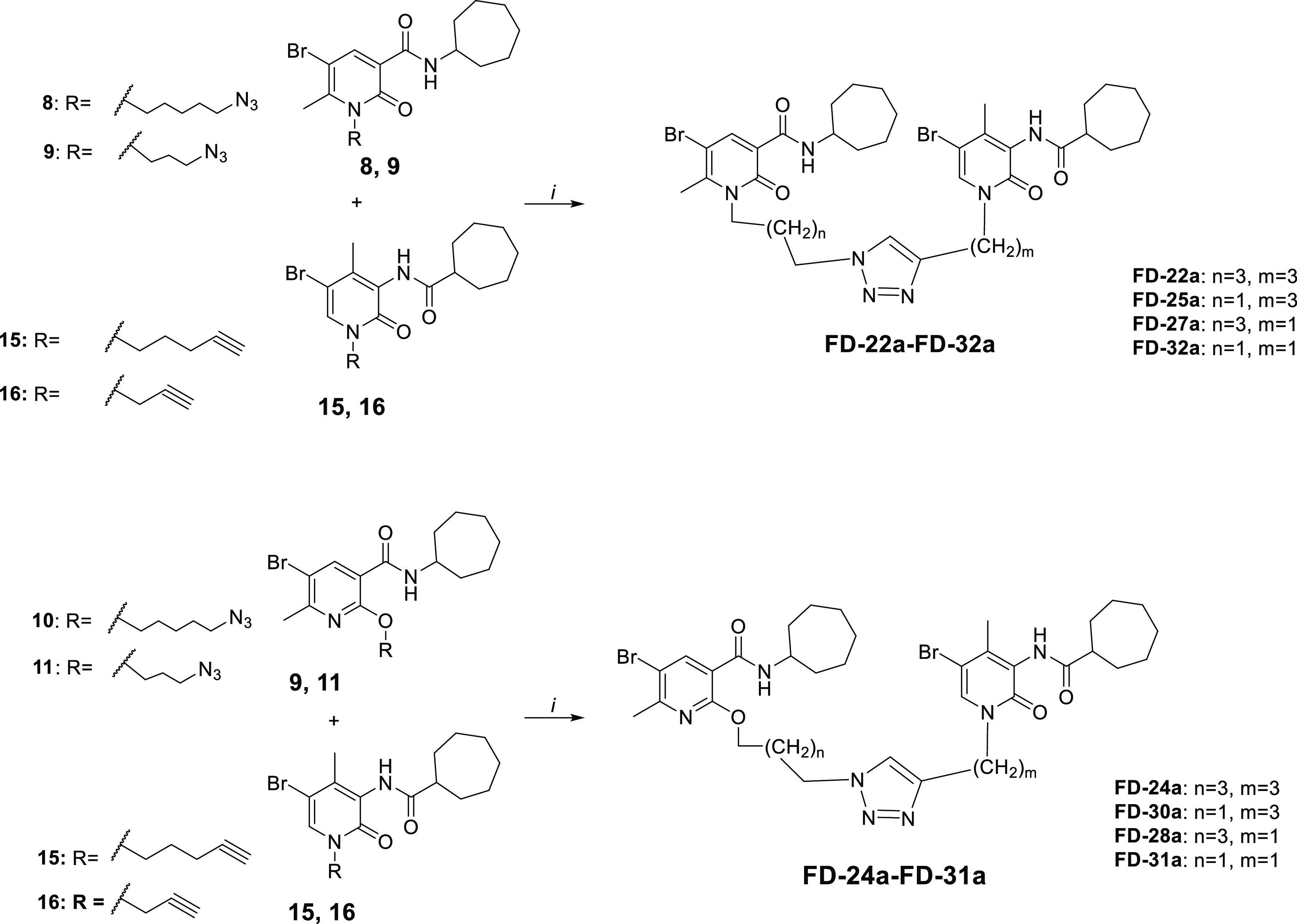
Synthetic Pathway for the Synthesis of Compounds **FD-22a**-**FD-31a** Reagents and conditions: (i) CuSO_4_·5H_2_O, sodium ascorbate, DMF/H_2_O 4:1, 80 °C, 2 h.

The synthesis of compounds **FD-22a**, **FD-25a, FD-27a,** and **FD-32a** was accomplished as depicted in Schemes 1–3. As described in Scheme 1, the methyl ester **1** was synthesized from the commercially available 6-methyl-2-*oxo*-1,2-dihydropyridine-3-carboxylic acid by heating in concentrated sulfuric acid and methanol at 90 °C for 24 h. Subsequently, a mixture of compound **1** and cycloheptylamine was heated in a sealed tube at 100 °C for 24 h to obtain the carboxamide derivative **2**, which was subjected to a bromination reaction on 5-position of the pyridine nucleus with Br_2_ in CHCl_3_, to afford compound **3**. Compound **3** was first treated with cesium fluoride in anhydrous DMF at room temperature for 1 h and then with 1,5-dibromopenthane or 1,3-dibromopropane at 30 °C for 12 h, affording the desired *N*-alkylated derivatives **4** and **5,** respectively, together with the corresponding O-substituted derivatives **6** and **7**. The two structural isomers were purified by flash chromatography. The corresponding azido derivatives **8-11** were synthesized by treatment of compounds **4**–**7** with sodium azide at 60 °C for 12 h in anhydrous DMF.

As reported in Scheme 2, the commercially available starting material 2-hydroxy-4-methyl-3-nitropyridine was treated with iron powder and ammonium chloride at 80 °C for 3 h to afford the amine compound **12**. The reaction between the amine derivative **12** and the cycloheptanecarbonyl chloride in DMF and triethylamine initially at 0 °C and then at room temperature for 24 h gave the amides **13**. The acyl chloride was prepared by a reaction between cycloheptanecarboxylic acid and oxalyl chloride at room temperature for 30 min. The 5-bromo derivative **14** was obtained from derivative **13** by treatment with Br_2_ in CHCl_3_ at room temperature for 12 h. Finally, compound **14** was subjected to a *N*-alkylation reaction by treatment with cesium fluoride in anhydrous DMF at room temperature for 1 h and then with the suitable halogenated reagent at 50 °C for 12 h, affording the desired alkyne derivatives **15** and **16**.

As illustrated in Scheme 3, the final compounds **FD-22a, FD-25a, FD-27a,** and **FD-32a** were easily obtained by a click chemistry reaction of the azido derivatives **8** and **9** with the alkyne derivatives **15** and **16** in DMF and water in the presence of CuSO_4_·5H_2_O and sodium ascorbate at 80 °C for 2 h. The same click reaction was also conducted between the azido derivatives **10** and **11** with the alkyne derivatives **15** and **16** to afford the compounds **FD-24a, FD-28a, FD-30a,** and **FD-31a.**

### Inhibition of Forskolin-Stimulated cAMP and Recruitment of βarrestin2

**Figure 2:**
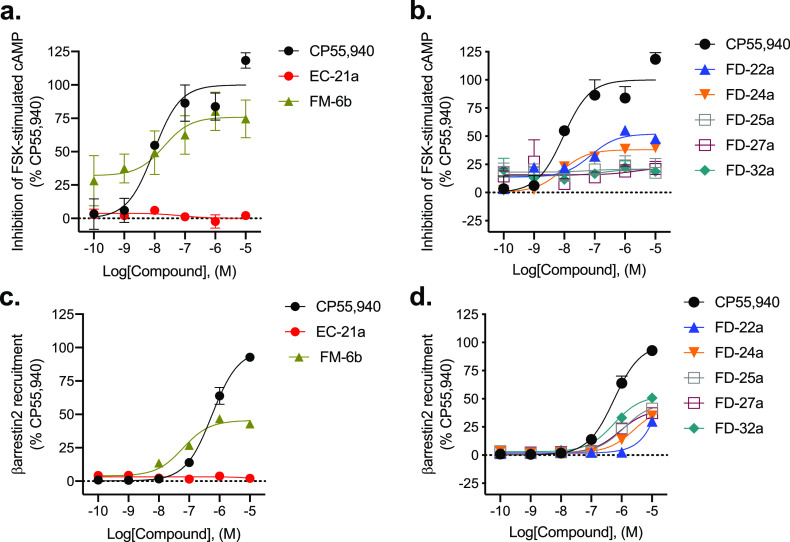
CB2R-dependent inhibition of FSK-stimulated cAMP and CB2R-dependent recruitment of βarrestin2. CHO cells stably expressing *h*CB2R were treated with 0.10 nM–10 μM compounds for 90 min, and cAMP inhibition (a,b) or βarrestin2 recruitment (c,d) was measured. cAMP and βarrestin2 recruitment data are expressed as the % CP55,940 response. Data were fitted to a nonlinear regression (three-parameter model, GraphPad v. 9.0). Data are mean ± S.E.M. of 6 independent experiments performed in triplicate. Data from these graphs is presented in Table 1. Statistical data for these graphs are presented in Table S1.

The new compounds **FD-22a**, **FD-25a, FD-27a, FD-32a**, the parent compounds **EC-21a** and **FM-6b,** and one of the *O*-alkylated derivatives, **FD-24a**, were characterized in an assay measuring the Gα_i/o_ protein-dependent inhibition of forskolin (FSK)-stimulated cAMP accumulation in CHO cells stably expressing *h*CB2R (Figure 2a,b). The nonselective orthosteric CBR ligand CP55,490 was used as a reference compound. Cells were treated with 10 μM FSK and CP55,490 or compound for 90 min to assess compound concentration-dependent activity (Figure 2a,b and Table 1).

**Table 1:** Inhibition of Forskolin-Stimulated cAMP and βarrestin2 Recruitment^a^

	inhibition of cAMP		βarrestin2 recruitment	
compound(s)	EC_50_ (nM) (95% C.I.)	*E*_max_ (% CP55, 940) ± S.E.M	EC_50_ (nM) (95% C.I.)	*E*_max_ (% CP55, 940) ± S.E.M
CP55, 940	9.4 (3.4–29)	100 ± 6.4	560 (410–760)	100 ± 3.4
EC-21a	>10,000	2.5 ± 0.53****	>10,000	1.4 ± 0.96****
FM-6b	20 (10–150)	76 ± 10***	63 (36–100)*	45 ± 1.5****
FD-22a	73 (14–230)	52 ± 3.4****	>10,000	30 ± 1.9****
FD-24a	8.0 (5.1–13)	38 ± 1.0****	>10,000	43 ± 2.3****
FD-25a	>10,000	21 ± 6.0****	>10,000	46 ± 1.6****
FD-27a	>10,000	22 ± 1.8****	>10,000	41 ± 1.1****
FD-32a	>10,000	21 ± 0.62****	560 (400–760)	53 ± 1.4****

a CB2R activity was quantified for cAMP inhibition using the DiscoveRx HitHunter assay (CHO *h*CB2R) in cells treated with compounds for 90 min and for βarrestin2 recruitment using the DiscoveRx PathHunter assay (CHO *h*CB2R) in cells treated with compounds for 90 min. Data were fit to a variable slope (three-parameter) nonlinear regression in GraphPad (v. 9). Data are mean with 95% confidence interval (C.I.) (EC_50_) or mean ± S.E.M, *n* = 6 independent experiments performed in triplicate. Statistical analyses were by nonoverlapping C.I. (EC_50_) or two-way ANOVA followed by Dunnett’s posthoc test (*E*_max_, Table S1). *p < 0.05, ***p < 0.001, ****p < 0.0001 relative to CP55,940 within assay. Data from this Table is graphed in Figure 2. Statistical data for these graphs are presented in Table S1.

Regarding the parent compounds, for **EC-21a**, no response was detected in the cAMP inhibition assay in accordance with its allosteric nature, while **FM-6b** showed high potency (20 [10–150] nM) and efficacy (76 ± 10%).

Among the **FD** compounds tested, **FD-22a** and **FD-24a** were the most interesting. **FD-22a** showed the highest efficacy (52 ± 3.4%) with nM potency (73 [14–230] nM), while the corresponding *O*-alkylated derivative **FD-24a** showed the highest potency (8.0 [5.1–13] nM) (Table 1). The other tested compounds **FD-25a**, **FD-27a**, and **FD-32a** showed very low potency and efficacy. This assay demonstrated that **FD-22a** and **FD-24a** are able to inhibit FSK-stimulated cAMP accumulation by *h*CB2R activation. Inhibition of FSK-stimulated was also quantified in CHO–K1 cells not expressing *h*CB2R because **FM-6b** and the **FD** compounds produced an elevated baseline response that may be the result of nonspecific activity (Figure S1). Treatment of CHO–K1 cells with 10 μM FSK elevated cAMP levels, and this cAMP accumulation was not altered by 10 μM CP55,940, as expected in cells without *h*CB1R or *h*CB2R (Figure S1). **FM-6b, FD-22a, FD-24a**, and to a lesser extent **EC-21a**, all inhibited cAMP accumulation in CHO–K1 cells (Figure S1). Therefore, the cAMP-modulatory effects of these compounds are not purely attributable to *h*CB2R, and future studies should identify the alternative targets for these ligands.

In addition to G protein-mediated signaling, GPCRs also interact with βarrestins, which facilitate receptor internalization, recycling, degradation, and signaling. **FD-22a**, **FD-24a**, **FD-25a**, **FD-27a**, and **FD-32a** and the parent compounds **EC-21a** and **FM-6b** were evaluated for their ability to enhance βarrestin2 recruitment in CHO cells stably expressing *h*CB2R. Cells were treated with CP55,490 or compound for 90 min (Figure 2c,d and Table 1). Regarding parent compounds, for **EC-21a**, no response was detected in accordance with its allosteric nature; **FM-6b** displayed both efficacy (45 ± 1.5%) with potency (63 [36–100] nM) for βarrestin2 recruitment. Although ligand bias was not estimated for **FM-6b**, this parent agonist was both more potent and more efficacious in the cAMP inhibition assay than the βarrestin2 recruitment, indicating that the parent agonist may display functional selectivity for Gα_i/o_-dependent signaling. **EC-21a** has not previously been shown to display a ligand bias,^19^ and in the present study its absence of activity alone makes estimates of ligand bias for the compound impracticable. Among the new derivatives, only **FD-32a** showed greater activity in the βarrestin2 assay relative to the cAMP inhibition assay. Conversely, for **FD-22a**, **FD-24a**, **FD-25a**, and **FD-27a**, no significant enhancement of βarrestin2 recruitment was observed (i.e., EC_50_ > 10,000 nM), whereas each of these compounds did display activity in the cAMP inhibition assay. Recent discussions regarding best practices in ligand bias estimation caution against applying operational models to allosteric ligands, let alone bitopic ligands.^40^ Based on these data, **FD-22a**, **FD-24a**, **FD-25a**, and **FD-27a** all display a trend toward Gα_i/o_-dependent signaling relative to βarrestin2 recruitment, which is consistent with their parent agonist, **FM-6b.** The increased βarrestin2 recruitment observed for **FD-32a** appears to be an emergent property of that compound.

**Figure 3:**
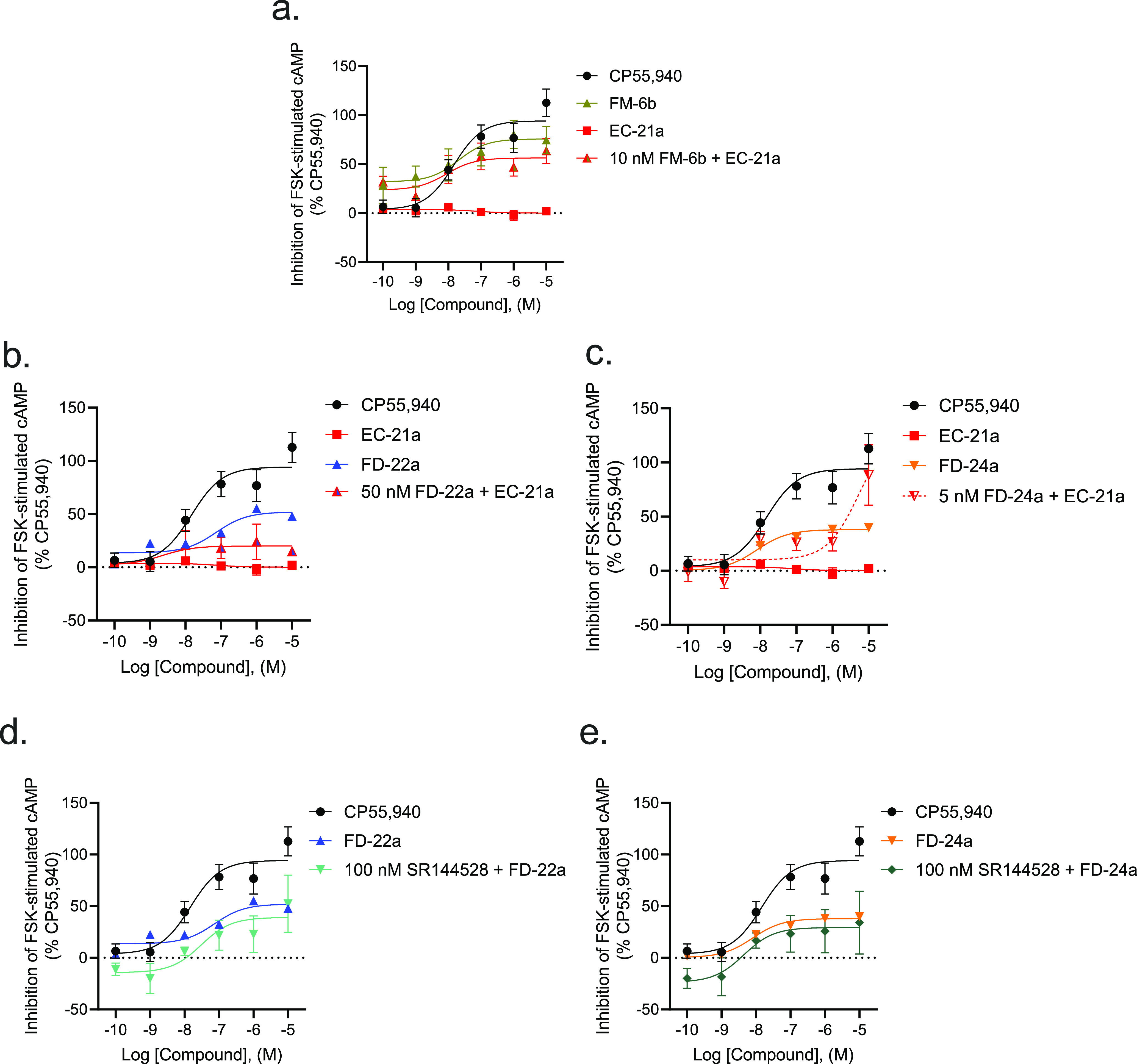
CB2R-dependent inhibition of FSK-stimulated cAMP CHO cells stably expressing *h*CB2R. cAMP inhibition data are expressed as the % CP55,940 response. Cells were treated with ligands simultaneously as indicated. 10 nM **FM-6b** (a), 50 nM **FD-22a** (b), and 5 nM **FD-24a** (c) were chosen after the completion of preliminary experiments with compounds alone for ease of calculations to approximate the EC_50_ for each compound alone. Addition of 100 nM SR144528 to 0.1 nM–10 μM of **FD-22a** (d) or of **FD-24a** (e). Data were fitted to a nonlinear regression (three-parameter model, GraphPad v. 9.0). Data are mean ± S.E.M. of 3–6 independent experiments performed in triplicate. Data from these graphs is presented in Table 2. Statistical data for these graphs are presented in Table S2.

Subsequently, we evaluated the effect of the combination of 10 nM **FM-6b** and 0.1 nM–10 μM of **EC-21a** on the inhibition of FSK-stimulated cAMP accumulation (Figure 3a and Table 2). 10 nM **FM-6b** in the presence of 0.1 nM **EC-21a** (Figure 3a, red and gold triangles) produced a cAMP inhibition response of 24 ± 8.7% (*E*_min_); and when 10 nM **FM-6b** was combined with increasing concentrations of **EC-21a**, cAMP inhibition increased up to 56 ± 7.0% at 10 μM **EC-21a** (*E*_max_; *p* = 0.0153, unpaired *t*-test [*t* = 2.921, df = 10]). This result is congruent with a positive allosteric behavior of **EC-21a** for the CB2R orthosteric agonist **FM-6b**.

**Table 2:** Inhibition of Forskolin-Stimulated cAMP^a^

compound(s)	EC_50_ (95% CI) (nM)	*E*_max_ ± SEM (%)
CP55,940	9.4 (3.4–29)	100 ± 6.4
FM-6b	20 (10–150)	76 ± 10***
EC-21a	>10,000	2.5 ± 0.53****^^^^
10 nM FM-6b + EC-21a	8.8 (0.62–19.7)	56 ± 7.0**
FD-22a	73 (14–230)	52 ± 3.4***†††
50 nM FD-22a + EC-21a	2.2 (0.98–31)	20 ± 4.9****
100 nM SR144528 + FD-22a	61 (47–170)*	39 ± 11****
FD-24a	8.0 (5.1–13)	38 ± 1.0****^^^††
5 nM FD-24a + EC-21a	>10,000	88 ± 17††††
100 nM SR144528 + FD-24a	6.5 (2.9–16)	29 ± 11****

a CB2R activity was quantified for cAMP inhibition using the DiscoveRx HitHunter assay (CHO *h*CB2R) in cells treated with compounds for 90 min. Data were fit to a variable slope (three-parameter) nonlinear regression in GraphPad (v. 9.0). Data are mean with 95% confidence interval (C.I.) (EC_50_) or mean ± S.E.M, *n* = 3–6 independent experiments performed in triplicate. Cells were treated with ligands simultaneously as indicated. 10 nM **FM-6b**, 50 nM **FD-22a**, and 5 nM **FD-24a** were chosen after the completion of preliminary experiments with compounds alone for ease of calculations to approximate the EC_50_ for each compound alone. *p < 0.05, **p < 0.01, ***p < 0.001, ****p < 0.0001 compared to CP55,940, ^^*p* < 0.01, *^^^^p* < 0.0001 compared to **FM-6b**, ††*p* < 0.01, †††*p* < 0.001, ††††*p* < 0.0001 compared to **EC-21a**, as determined by nonoverlapping 95% C.I. (EC_50_) or one-way ANOVA followed by Tukey’s posthoc test (*E*_max_). Data from this Table are graphed in Figure 3. Statistical data for these graphs are presented in Table S2.

We also evaluated the effect of the combination of EC_50_**FD-22a** with 0.1 nM–10 μM **EC-21a** on the inhibition of FSK-stimulated cAMP accumulation (Figure 3b and Table 2). The results indicate that the activity of **FD-22a** is not significantly increased by the PAM **EC-21a**. If **FD-22a** were a pure orthosteric agonist, we might expect **EC-21a** to enhance its activity more than it does (as for 10 nM **FM-6b**). The observation, though, is that no significant enhancement occurred, and this could indicate that **FD-22a** and **EC-21a** share the same allosteric site.

The same assay was conducted for the combination of EC_50_**FD-24a** with 0.1 nM–10 μM **EC-21a.** The results (Figure 3c and Table 2) indicate that high concentrations of **EC-21a** greatly increase the agonist activity of **FD-24a** but not at lower concentrations. The obtained data suggest that if **FD-24a** is binding an allosteric site, **FD-24a** and **EC-21a** compete for that allosteric site until sufficiently high concentrations of **EC-21a** are achieved. At those high concentrations, **EC-21a** facilitates the agonism of **FD-24a** to the high levels observed.

Finally, we tested 0.1 nM–10 μM **FD-22a** and **FD-24a** compounds both against 100 nM CB2R antagonist/inverse agonist SR144528 (Figure 3d,e and Table 2). The results showed that as the concentration of **FD-22a** or **FD-24a** is increased, SR144528 is competed from the orthosteric site of the receptor, effectively removing any of the antagonism/inverse agonism caused by 100 nM SR144528. Unexpectedly, the addition of 100 nM SR144528 did not produce a rightward dextral shift in the **FD-22a** and **FD-24a** concentration–response curves (Figure 3d,e). This lack of effect may represent the contribution of the allosteric ligand. Importantly, the concentration-dependent inhibition of cAMP support the activity of these compounds at *h*CB2R (Figure S1). Taken together, these data support that **FD-22a** and **FD-24a** potentially interact with both orthosteric and allosteric sites (i.e., bitopic). However, the data suggest that **FD-24a** has a lower affinity for the allosteric site and a higher affinity for the orthosteric site than **FD-22a** because at high concentrations of **EC-21a**, the curve for EC_50_**FD-24a** + **EC-21a** approaches 100%.

### [^**3**^H]CP55,940 Binding Assays

**Figure 4:**
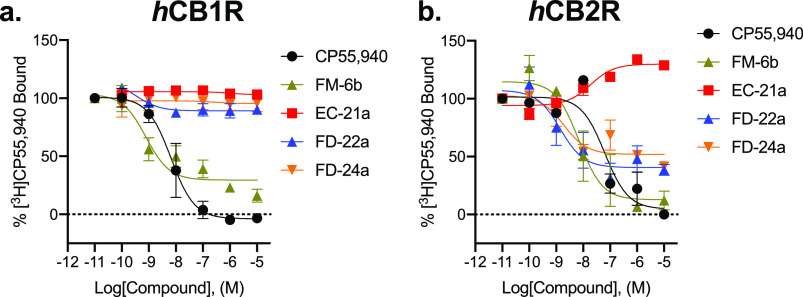
[^3^H]CP55, 940 binding to CB1R (a) and CB2R (b). Membranes from CHO cells stably expressing *h*CB1R or *h*CB2R were treated with 1 nM [^3^H]CP55,940 and 0.10 nM–10 μM compounds for 2 h. Data are expressed as %[^3^H]CP55,940 bound. Data were fitted to a nonlinear regression (three-parameter model, GraphPad v. 9.0). Data are mean ± S.E.M. of 3 independent experiments performed in duplicate. Data from these graphs is presented in Table 3. Statistical data for these graphs are presented in Table S3.

Following characterization of G protein-mediated cAMP inhibition and βarrestin2 recruitment, we assessed ligand affinity for **FM-6b**, **EC-21a**, **FD-22a**, and **FD-24a** at *h*CB1R and *h*CB2R using a [^3^H]CP55,940 radioligand displacement assay using membranes derived from CHO cells stably expressing either receptor. At *h*CB1R, **FM-6b** was able to displace [^3^H]CP55, 940 in accordance with the existing literature,^34^ but none of the other compounds tested did so, indicating little to no affinity of these ligands at *h*CB1R (Figure 4a and Table 3). The displacement of [^3^H]CP55, 940 from *h*CB1R by **FM-6b** was irregular with an *E*_min_ > 0, suggesting **FM-6b** may exhibit differential affinity for *h*CB1R in active versus inactive conformations; however, such experiments are beyond the scope of the present study. Regarding the lack of [^3^H]CP55, 940 displacement from *h*CB1R by **FD-22a** and **FD-24a**, it is possible that the addition of a linker and *h*CB2R-specific PAM **(EC-21a**) to **FM-6b** prevented these compounds from effectively binding *h*CB1R and thus reducing their observable affinity to zero. However, additional future experiments are necessary to determine the mechanisms of receptor subtype specificity for these bitopic ligands. At *h*CB2R, **EC-21a** augmented [^3^H]CP55,940 bound to the receptor, consistent with its activity as a CB2R PAM (Figure 4b and Table 3). **FM-6b** fully displaced [^3^H]CP55,940 from *h*CB2R, consistent with its orthosteric agonist mode of action (Figure 4b and Table 3) and in accordance with earlier data.^34^**FD-22a** and **FD-24a** both partially displaced [^3^H]CP55,940 from *h*CB2R, although their incomplete displacement indicates more complex pharmacology than a typical orthosteric agonist (Figure 4b and Table 3). It is possible that the PAM and agonist moieties of these compounds produce opposing effects on [^3^H]CP55,940 binding that need to be cautiously interpreted when considering the mechanism(s) of bitopic ligands.

**Table 3:** [^3^H] CP55,940 Binding^a^

	*h*CB1R		*h*CB2R	
compound	*K*_i_ (nM) (95% C.I.)	*E*_min_ (% CP55, 940) ± S.E.M	*K*_i_ (nM) (95% C.I.)	*E*_min_ (% CP55,940) ± S.E.M
CP55, 940	6.6 (2.7–15)	0.0 ± 5.6	34 (2.7–57)	0.0 ± 8.3
EC-21a	>10,000	103 ± 2.5****	19 (4.5–46)	130 ± 3.1****
FM-6b	0.79 (0.23–4.3)	29 ± 4.7**	7.0 (1.9–26)	13 ± 8.2
FD-22a	>10,000	89 ± 2.8****	1.4 (0.27–11)	40 ± 6.4***
FD-24a	>10,000	95 ± 7.1****	1.9 (0.43–9.8)	52 ± 4.6****

a Competition binding of [^3^H]CP55,940 to CB1R and CB2R was quantified in CHO *h*CB1R or CHO *h*CB2R cell membranes incubated with compounds for 2 h. Data were fit to a three-parameter nonlinear regression in GraphPad (v. 9.0). Data are mean with 95% C.I. (*K*_i_) or mean ± S.E.M, *n* = 3 independent experiments performed in duplicate. Statistical analyses were by nonoverlapping C.I. or two-way ANOVA followed by Dunnett’s posthoc test. **p < 0.01, ***p < 0.001, ****p < 0.0001 relative to CP55, 940 within receptor. Data from this Table are graphed in Figure 4. Statistical data for these graphs are presented in Table S3.

### Microglial Cell Inflammatory Models

Given the promising results previously reported regarding parent compounds **FM-6b** and **EC-21a** activity against LPS-stimulated neuroinflammation in BV2 microglial cells,^37^ we initially decided to use the same experimental model to investigate the anti-inflammatory properties of the novel compounds **FD-22a** and **FD-24a** emerged from functional studies as the most active of the series. Therefore, 1 μM test compounds (**FD-22a** or **FD-24a**) were administered to BV2 cells 1 h before LPS treatment, and the release of pro- e anti-inflammatory markers, such as interleukin-6 and 10 (IL-6 and IL-10), was measured by using ELISA assays. Furthermore, to demonstrate a link between test compound’s anti-inflammatory activity and CB2R activation, a simultaneous treatment with CB2R antagonist SR144528 was also performed.

**Figure 5:**
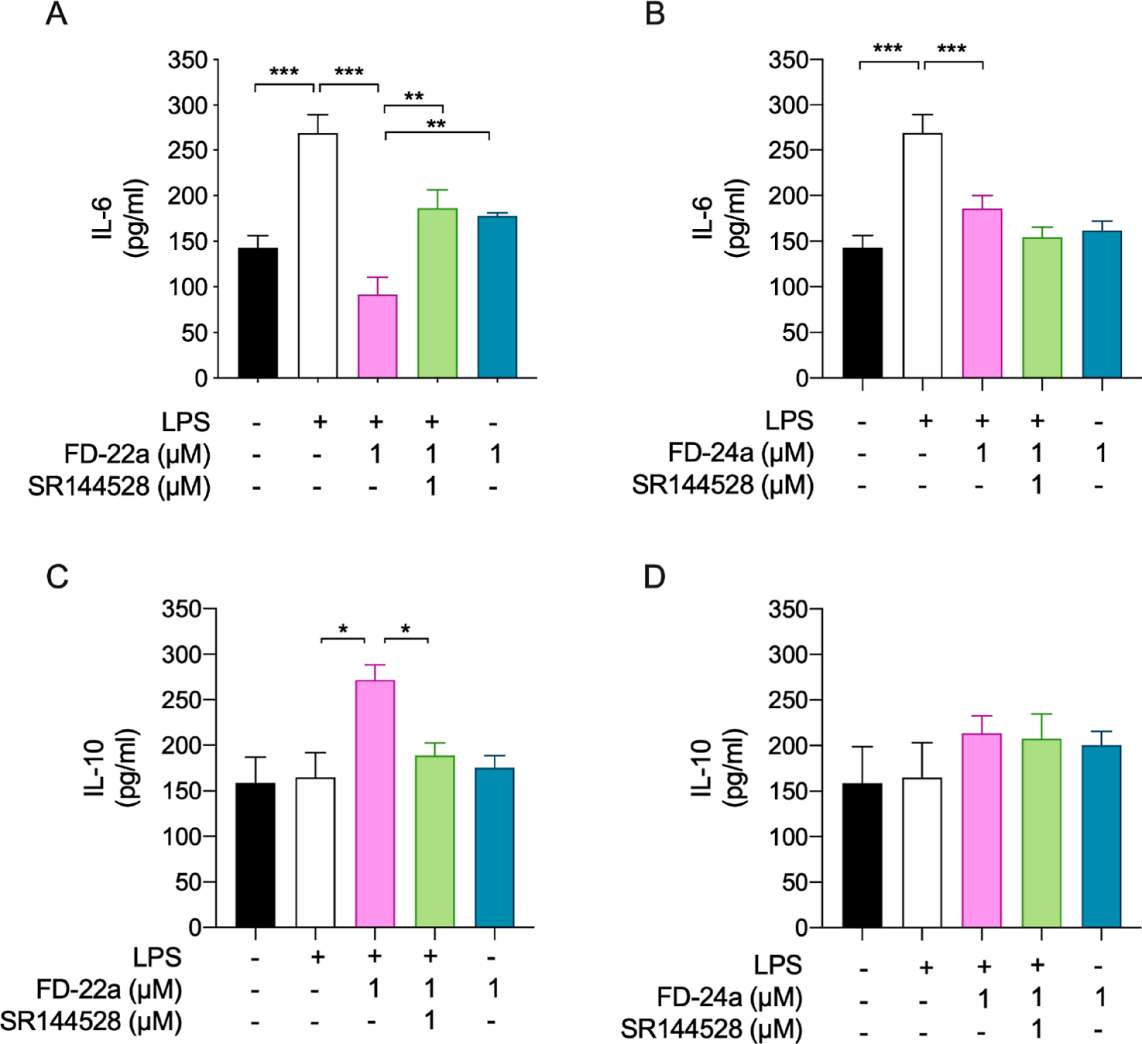
Ability of **FD-22a** (A,C) and **FD-24a** (B,D) to decrease the inflammatory phenotype of LPS-stimulated BV2 microglial cells by the modulation of CB2R. Bars represent the release (pg/mL) of ILs in the presence of the drugs. Data represent mean ± (bars) from *n* = 3 independent experiments performed in duplicate. Statistical analysis was performed by ordinary one-way ANOVA followed by Tukey’s multiple comparison test. *p < 0.05, **p < 0.01 and ***p < 0.001.

As shown in Figure 5, the exposure of BV2 cells to LPS induced a significant increase of pro-inflammatory IL-6 release as compared to control cells, while no effect on anti-inflammatory IL-10 release was observed. Among the novel compounds tested, **FD-22a** revealed to efficiently prevent the inflammatory response induced by an LPS stimulus, as shown by the observed significant decrease of IL-6 release (Figure 5A) and concomitant increase of IL-10 release (Figure 5C). Notably, the modulatory activity of **FD-22a** on both IL-6 and IL-10 release totally reverted in the presence of CB2R antagonist SR144528 (1 μM), suggesting CB2R to be the exclusive target for the **FD-22a**-mediated effects observed in our experimental settings.

**Figure 6:**
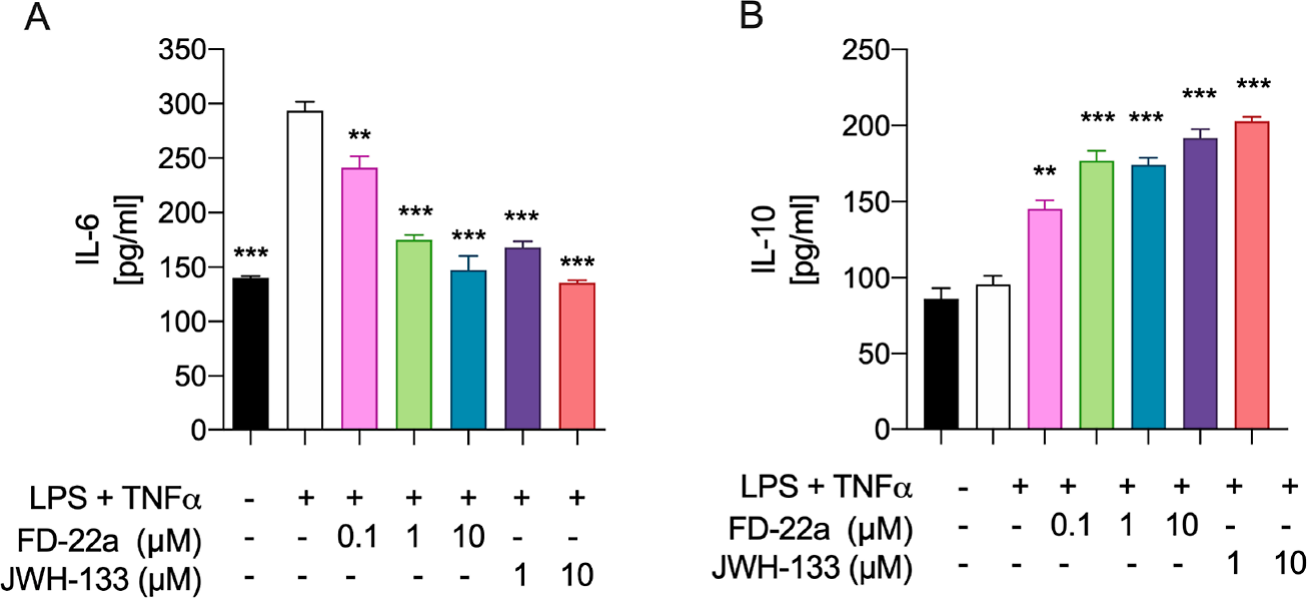
Release of inflammatory (IL-6) (A) and anti-inflammatory (IL-10) (B) interleukins induced by different concentrations of **FD-22a**. Data represent means ± S.E.M. from *n* = 3 independent experiments performed in duplicate. The selective CB2R agonist JWH133 was used as positive control. Statistical analysis was performed by ordinary one-way ANOVA followed by Tukey’s multiple comparison test. **p < 0.01 and ***p < 0.001 compared to cells treated with LPS and TNFα.

**Figure 7:**
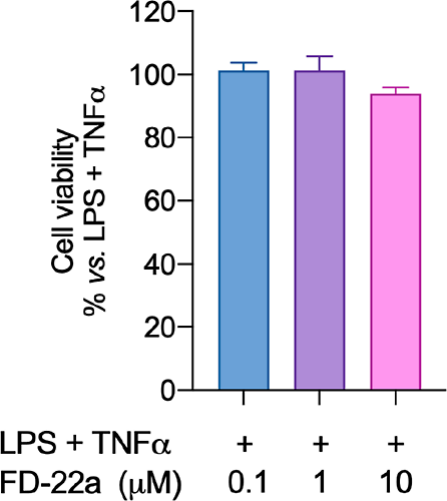
MTT assay performed with different concentrations of **FD-22a.** Data represent means ± S.E.M. from *n* = 3 independent experiments performed in triplicate. Statistical analysis was performed by ordinary one-way ANOVA followed by Tukey’s multiple comparison test.

To further evaluate the potential of **FD-22a** to prevent neuroinflammation, we tested this compound on a human microglial cell inflammatory model. The human microglia display important biochemical and pharmacological differences compared to rodent microglia,^41^ and HMC3 cells may provide a model of human microglial inflammation that can be used in preclinical screening of promising compounds. Therefore, we first set up a human model of microglial inflammation by exposing HMC3 cells to an LPS/TNFα stimulus.^42^ In agreement with our previous observations,^37^xposure of HMC3 cells to the LPS/TNFα stimulus resulted in a significant increase of pro-inflammatory IL-6 release in cell media, while no effect on anti-inflammatory IL-10 release was observed as compared with control cells (Figure 6). Then, dose–response experiments were carried out by exposing HMC3 cells to pretreatment with increasing concentrations of **FD-22a** (100 nM, 1 μM, and 10 μM), followed by LPS/TNFα treatment for 24 h. Measurements of IL-6 and IL-10 levels in cell media by ELISA (Figure 6) revealed that when used at 1 μM concentration, compound **FD-22a** displayed a significant anti-inflammatory activity in the absence of any relevant cytotoxic effect as assessed by the MTT reduction assay (Figure 7).

**Figure 8:**
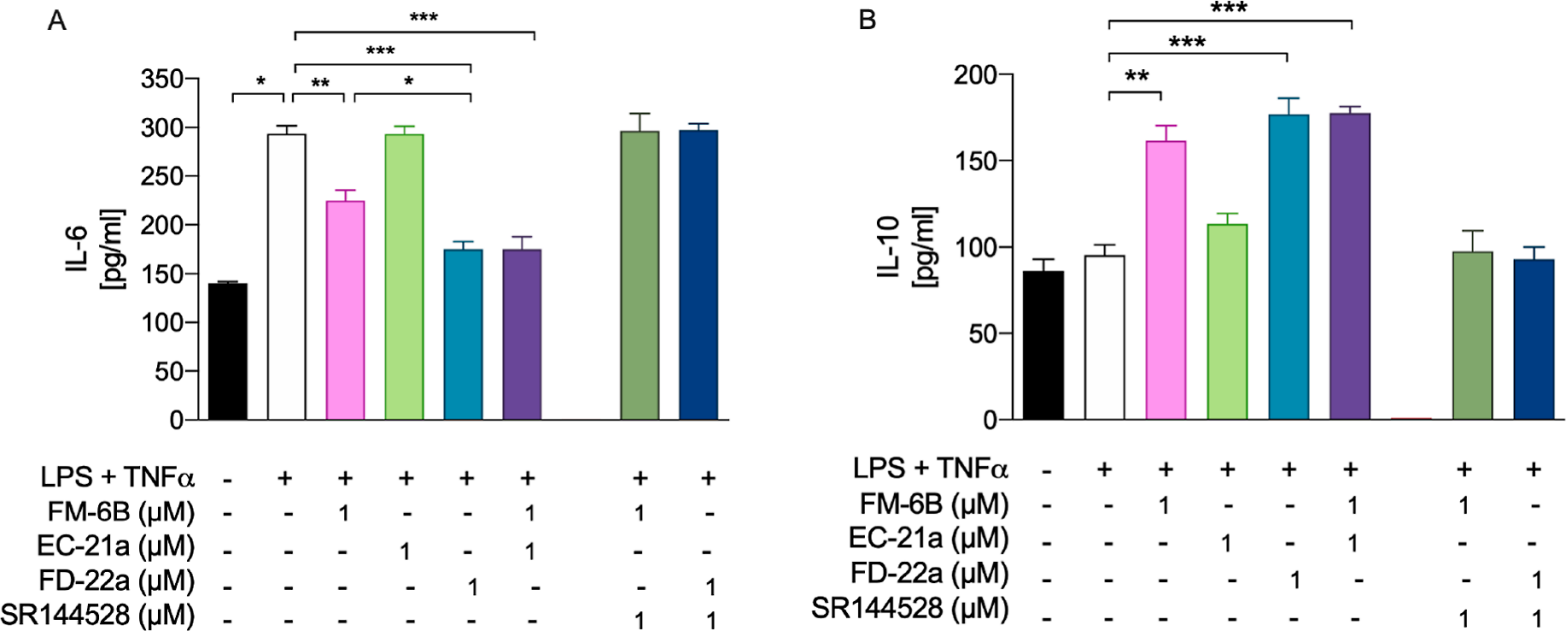
Ability of **FD-22a** to decrease the inflammatory phenotype of LPS + TNFα–stimulated HMC3 by the modulation of CB2R. Bars represent the release (pg/mL) of IL-6 (A) and IL-10 (B) in the presence of the drugs at the indicated concentrations. Data represent means ± S.E.M. from *n* = 3 independent experiments performed in duplicate. Statistical analysis was performed by ordinary one-way ANOVA followed by Tukey’s multiple comparison test.*p < 0.05, **p < 0.01 and ***p < 0.001.

We finally performed a comparison between the anti-inflammatory capacity of **FD-22a** and that of parent compounds **FM-6b** and **EC-21a**. Allosteric/orthosteric CB2R ligands co-administration experiments were also performed. As shown in Figure 8, the bitopic compound **FD-22a** (1 μM) revealed a slightly better activity upon modulating both IL-6 and IL-10 release from HMC3 cells as compared to orthosteric analog **FM-6b** used at the same concentration. Notably, in our experimental setting, the anti-inflammatory activity of bitopic analog **FD-22a** was comparable to that observed in allosteric/orthosteric co-administration experiments [i.e., **EC-21a** (1 μM) + **FM-6b** (1 μM)], confirming its efficacy as a bitopic agent. Notably, pretreatment with CB2R selective antagonist SR144528 (1 μM) completely abolished the anti-inflammatory action of **FD-22a** and **FM-6b**, further confirming a CB2R-mediated anti-inflammatory effect in HMC3 microglial cells.

### Antinociceptive Effects of FD-22a in Animal Models of Neuropathic Pain

**Figure 9:**
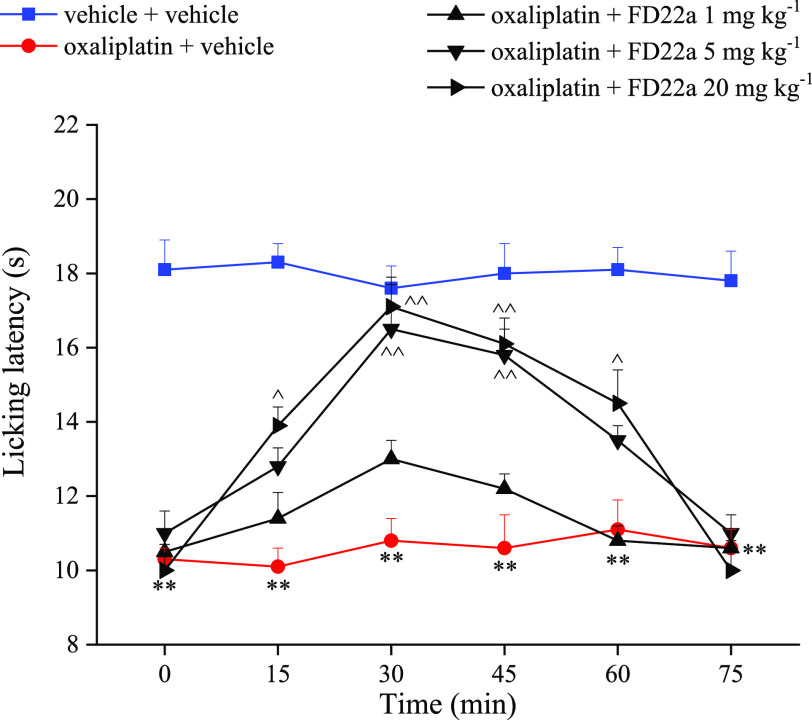
Effect of **FD-22a** on oxaliplatin induced neuropathic pain in mice. The response to a thermal stimulus was evaluated by the cold plate test measuring the latency (s) to pain related behaviors (lifting or licking of the paw). Mice were daily treated *i.p.* with oxp 2.4 mg kg^–1^. Tests were performed on day 15. **FD-22a** (1, 5, 20 mg kg^–1^) was *p.o.*, and measurements were performed 15, 30, 45, 60, and 75 min after injection. Control mice were treated with vehicle. Each value represents the mean of 16 mice per group performed in 2 different experimental sets. **p < 0.01 *vs* vehicle + vehicle treated mice.^*p* < 0.05 and ^^*p* < 0.01 *vs* oxaliplatin + vehicle treated mice.

**Figure 10:**
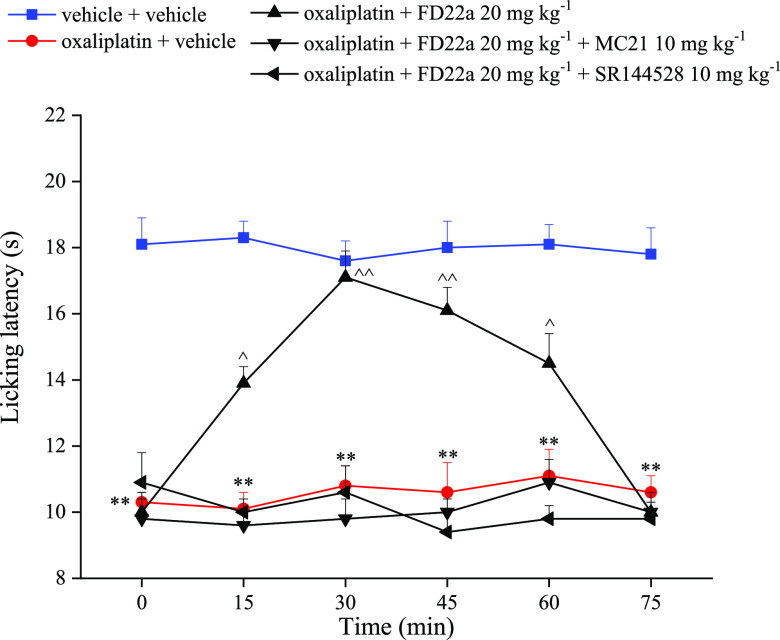
Effects of CB2 antagonism on **FD-22a** pain relieving efficacy. The response to a thermal stimulus was evaluated by the cold plate test measuring the latency (s) to pain-related behaviors (lifting or licking of the paw). Mice were daily treated *i.p*. with oxaliplatin 2.4 mg kg^–1^. Tests were performed on day 15. The selective CB2R antagonists MC21 and SR144528 (10 mg kg^–1^) were administered i.p. 15 min before **FD-22a** (20 mg kg^–1^*p.o.*). Measurements were performed 15, 30, 45, 60, and 75 min after the injection of **FD-22a**. Control mice were treated with a vehicle. Each value represents the mean of 16 mice per group performed in 2 different experimental sets. **p < 0.01 *vs* vehicle + vehicle treated mice ^p < 0.05 and ^^p < 0.01 *vs* oxaliplatin + vehicle treated mice.

Neuropathic pain may be induced by traumatic injury, metabolic challenges, and chemotherapeutic agents. Pharmacotherapies used to treat neuropathic pain produce inadequate pain relief and/or unwanted side effects. Thus, the identification of novel therapeutic approaches with limited side effect profiles remains an urgent medical need. Cannabinoids suppress behavioral responses to noxious stimulation and suppress nociceptive transmission through activation of CB1R and CB2R. Moreover, CB2R is upregulated in CNS and dorsal root ganglia by pathological pain states, and CB2R was identified as a therapeutic target for treating pathological pain states. These observations prompted us to examine the ability of **FD-22a** to alleviate signs of neuropathic pain in a mouse model of nociceptive behavior caused by the chemotherapeutic agent, oxaliplatin. Daily treatment with the neurotoxic compound oxaliplatin (2.4 mg kg^–1^ intraperitoneally, *i.p.*) progressively decreased the pain threshold of mice evaluated as hypersensitivity to a cold non-noxious stimulus (allodynia-like measurements; cold plate).^43,44^ A single administration (*p.o.*) of **FD-22a** dose-dependently (1–20 mg kg^–1^) relieved neuropathic pain starting from the dose of 5 mg kg^–1^. The dose of 20 mg kg^–1^ was able to completely revert oxaliplatin-dependent allodynia. Efficacy onset and duration ranged between 15 and 60 min after treatment (Figure 9). Compound activity was comparable to that of well-known pain-relieving drugs pregabalin and duloxetine in the same model.^45^ Notably, the antinociceptive effect of **FD-22a** was higher than that of the parent CB2R orthosteric agonist **FM-6b.**(34) Furthermore, the role of CB2R in the antineuropathic effect of **FD-22a** was also studied by using the selective antagonists of CB2R, MC21,^46^ and SR144528. Oxaliplatin-induced hypersensitivity is maintained when **FD-22a** was administered in animals pretreated with antagonists, highlighting the pharmacodynamic relevance of CB2R in the pain-relieving effect of **FD-22a** (Figure 10).

### Cavity Identification

A first attempt to detect the possible CB2R allosteric binding site was the site search analysis performed on all available CB2R three-dimensional structures using FLAP software.^47,48^ Resulting cavities calculated in 5ZTY,^49^ 6KPC,^50^ 6KPF,^50^ and 6PT0^51^ crystallographic structures are reported in Figure S2. Excluding the cavity (not shown for clarity) relative to the Gi site, three cavities are conserved: the huge yellow orthosteric cavity S2, in common with all the activation states, the green one S2, among TM1, TM7, and TM8, and the orange colored S3, in the region between TM4 and TM5. We focused our attention especially on the active conformations of 6KPF and 6PT0 structures, which are consistent with PAMs binding and activity: in these proteins the S3 cavity extends from TM4 to TM5 in the middle of the lipidic region, and it partially overlaps the cholesterol-binding site of CLR404 in 6PT0.^51^ Furthermore, about the same region between TM4 and TM5 is the location of two cholesterol molecules in the active conformation of the CB1R:^52^ this is interesting considering that cholesterol is proposed to indirectly modulate the GPCR activation.^53^

### Docking

**Figure 11:**
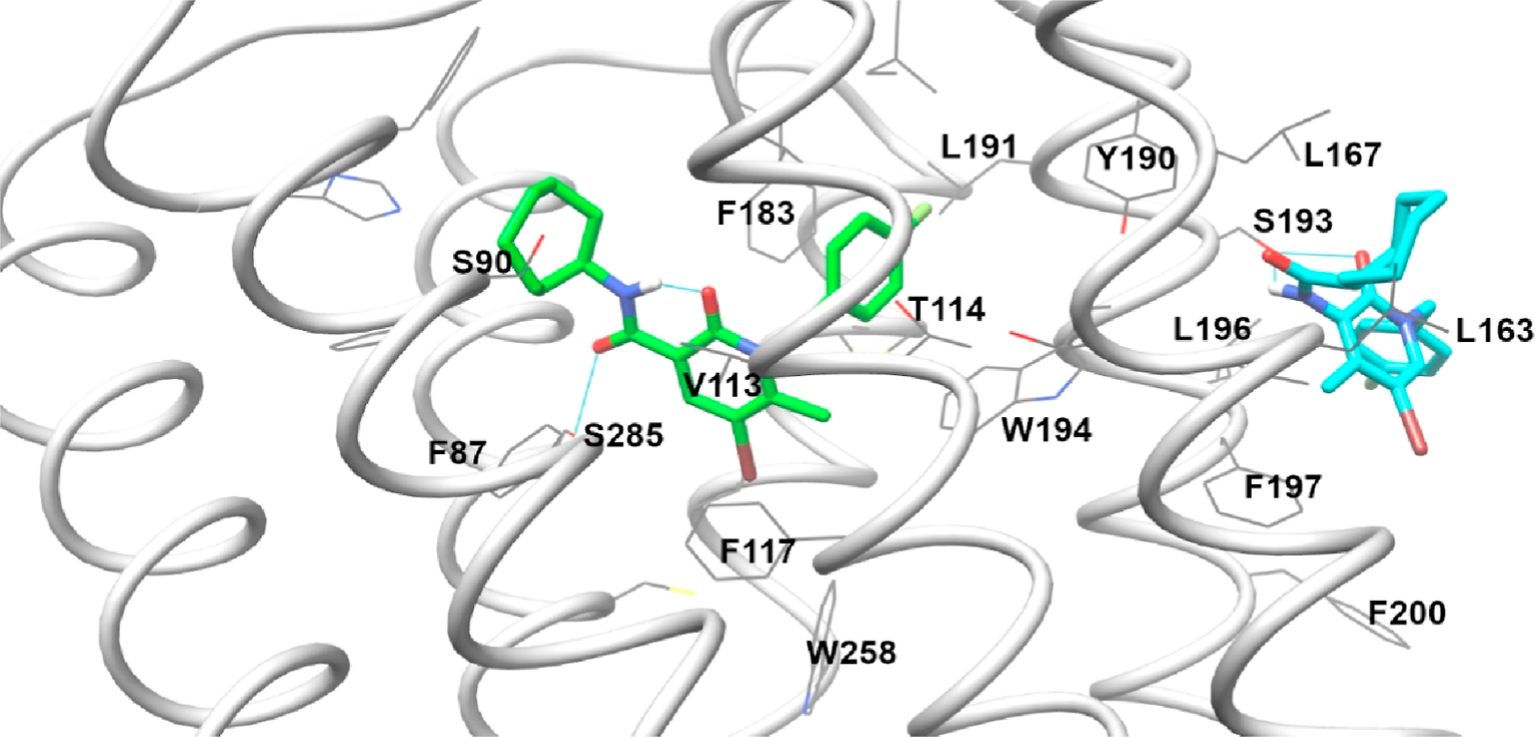
Results of **FM-6b** (green colored) and **EC-21a** (cyan colored) docking in the 6PT0 structure.

There are no pieces of evidence about the allosteric binding site of the CB2R. For the CB1R subtype, a crystal structure of the agonist-bound receptor in the presence of NAM ORG27569 was recently published,^54^ but it is unlikely that **EC-21a** and ORG27569 share the same site: the key residue Phe237 of the ORG27569-CB1 binding site is nonconserved in CB2R,^54^ and the biological effect is the opposite, **EC-21a** being a PAM.^35^ The orthosteric CB2R binding site is, on the contrary, well known now. So, we began the study with the docking of **FM-6b** in the agonist-bound structures, choosing 6PT0 as the active conformation structure because missing atoms or multiple conformations were absent.^51^ The **FM-6b** docking pose in the 6PT0 structure confirmed the one’s previously calculated, showing an interesting intramolecular hydrogen bond between the amidic NH and the carbonyl of 2-oxopyridine, which guarantees the coplanarity (see Figure 11).^34^

The second step of our study was the docking of **EC-21a** in CB2R. We performed the docking in the 6PT0 structure, in its agonist- and Gi– bound form consistently with **EC-21a** activity, using the orthosteric binding site occupied by the crystallographic ligand.^51^ All results are reported in Figure S3: potential binding sites are three, considering all the scoring function results.

One site is in the central cavity of the receptor, in a region comprised among the extracellular side of TM1, TM2, and TM7, included in the huge S1 cavity detected by FLAP around the orthosteric site (well visible in cyan poses of Figure S3a) and corresponding to the allosteric site suggested by Navarro *et al.* for some cannabidiol analogues^55^ and by Morales *et al.* for homobivalent bitopic ligands.^56^ Furthermore, it corresponds with “site H” revealed by Yuan *et al.*(57) The second potential site is on the surface of TM2, and it is partially coincident with the extroflexion of the S1 surface calculated by FLAP (Figure S3a–c). The third potential binding site is at the top of the S3 FLAP region (see Figures S3c and 12 for details on interactions), which corresponds with “site K” previously suggested by Yuan *et al.*(57) The distance between the pseudo center of the orthosteric ligand and the allosteric one in each of the three locations is 11.4, 16.4, and 18.5 Å, respectively. It is evident that the high correlation between receptor activation state and population results in the inactive form 5TZY (light green, Figure S3a) and the intermediate one 6KPC (dark green, Figure S3b), and the most populated sites are in the FLAP S1 cavity. In the active Gi-bound form 6PT0 (grey, Figure S3c), some poses are calculated around the loop surfaces and are not reported for clarity; the most populated poses are predicted through all fitness functions at the top of the FLAP S3 cavity on the receptor surface between TM4 and TM5. The best pose was reported in Figure 11: **EC-21a** engages many hydrophobic interactions with the CB2R surface and a hydrogen bond with Ser193.

**Figure 12:**
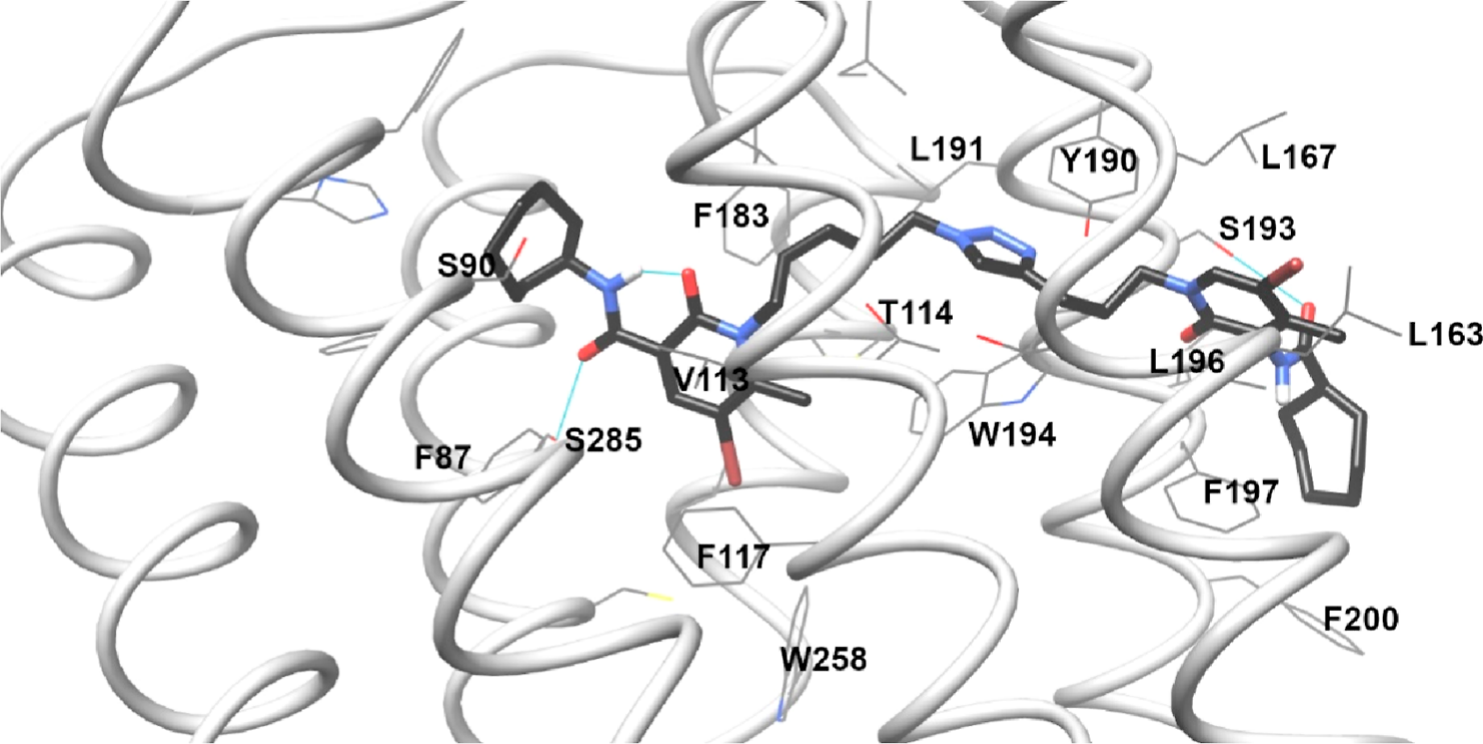
Results of **FD-22a** docking in the 6PT0 structure. Hydrogen bonds are reported as cyan lines.

The third step was the docking of the bitopic ligand **FD-22a** and all **FD** compounds. The standard procedure produced unreliable poses for **FD-22a**, which is too long for the S1 cavity and adopts a distorted conformation not matching the usual orthosteric ligand disposition. A scaffold match constraint was then applied during the docking calculation for simulating the rationale of the bitopic **FD-22a** design: the pharmacophoric portion of the CB2R orthosteric agonist **FM-6b** situated in the classic binding site linked to the pharmacophoric portion of the CB2R PAM **EC-21a**. Indeed, docking was performed, constraining the orthosteric portion of **FD-22a** on the **FM-6b** docking pose. The resulting pose is reported in Figure 12: the **FD-22a** linker guided the allosteric portion toward the S3 cavity, where it engages a hydrogen bond with Ser193, as **EC-21a.** Obviously, the pose of the allosteric tail is not exactly the same of **EC-21a** because **FD-22a** lacks of the fluorophenyl ring, and it is linked to the orthosteric site through a 11 atoms chain, a fixed length which just allows to reach the CB2R surface.

The distance analysis, previously reported, confirmed that the binding site coincident with the S3 cavity is the only one compatible with the **FD-22a** and **FD-24a** chain length, hypothesizing the contemporary interaction in the orthosteric site. The site comprised among the extracellular side of TM1, TM2, and TM7, already suggested as a potential allosteric CB2R binding site,^55−57^ which is distant 11.4 Å from the orthosteric one, is neither fitting with the **FD-22a** and **FD-24a** chain length nor with the bulk of heterocycle moieties in the interhelical space between TM1 and TM7. In fact, apart from **FD-22a** and **FD-24a**, all other docked compounds preferred to direct the allosteric tail toward the extracellular loops or between TM5 and TM6 using ASP, PLP, and CHEMSCORE fitness functions.^58^ Only GOLDSCORE has produced, for all compounds **FD-25a**, **FD-27a**, **FD-28a**, **FD-30a**, **FD-31a**, and **FD-32a**, the docking poses in the S3 cavity but with a steric clash increasing with shortening length of the spacer chain. In particular, we can highlight two key-moieties in the allosteric portion: the triazole ring and the amide. The triazole ring in both **FD-22a** and **FD-24a** (see Figures S4a and 12) lies between Tyr190 and Trp194, and the amide (both NH and/or C=O, in different docking poses) engages a hydrogen bond with Ser193. The influence of the oxygen in the **FD-24a** linker is very light: it affects the intramolecular hydrogen bond with the amide moiety, which is weaker with the ether group of **FD-24a** rather than the carbonyl of the 2-oxopyridine of **FD-22a**. In **FD-25a** and **FD-30a** (Figure S4b), the shorter n-linker (see Figure 1) avoids the right interaction of the triazole ring with Tyr190 and Trp194 and worsens the orthosteric disposition in spite of the docking constraint. Furthermore, the 2-*oxo*-pyridine moiety clashes with the helix surface. On the contrary, in **FD-27a** and **FD-28a** (Figure S4c), the n-linker allows the interaction of the triazole ring with Ty190 and Trp194 and partially preserves the orthosteric disposition (without any intramolecular hydrogen bond), but the m-linker is too short, and the 2-*oxo*-pyridine moiety clashes anyway on TM4 and TM5. In **FD-31a** and **FD-32a** (Figure S4d), the length *m* = 1 and *n* = 1 produces a distortion in the interaction of the orthosteric portion and a catastrophic clash of the 2-*oxo*-pyridine moiety on TM4 and TM5.

Further studies are requested to confirm this hypothesis, but it seems that the best m and n values must be calculated to guarantee the distance between the orthosteric portion and Tyr190/Trp194 and from the aromatic p-stacking and Ser193, respectively. Values of the m length less than 3 could induce steric clashes with TM surfaces.

The key residue Ser193 has been already analyzed through mutagenesis studies,^59^ being a no conserved residue, for exploring its role in agonist/antagonist binding. The substitution of serine with glycine resulted in not affecting the competition binding of CP 55, 940, SR 144528, and WIN 55212-2, but this is in agreement with our results because Ser193 does not interact with orthosteric ligands. Analyzing the residue context of the potential allosteric site shows that it is rich with no conserved residues between CB1R and CB2R (blue and grey in Figure S5); in particular, it is interesting to highlight that the conformation of Ser193 switches 180° between the Gi-bound conformation of 6KPF/6PT0- and unbound conformations of 5ZTY/6KPC- crystallographic structures (data not shown), and that from the region of S193 begin the helix disalignment between Gi-bound and unbound conformation of TM4 and TM5. This evidence supports the hypothesis of the presence of an allosteric site in this region, which accommodates cholesterol in both CB1R and CB2R. The pose of cholesterol in these receptors is slightly different due to the high presence of unconserved residues in this area.

## Conclusions

We have presented evidence that the compounds **FD-22a** and **FD-24a** are the first CB2R heterobivalent bitopic ligands synthesized. To the best of our knowledge, only CB2R homobivalent bitopic ligands obtained using the same pharmacophore portion for both binding sites are reported in the literature.^56^ Bitopic GPCR ligands offer several theoretical advantages over purely orthosteric or allosteric ligands.^12,14^ Namely, bitopic ligands may display a greater receptor subtype selectivity and greater affinity to their receptor than either allosteric or orthosteric ligand alone, and bitopic ligands might be capable of promoting biased signaling beyond their allosteric or orthosteric components alone.^12,14^ In accordance with these advantages, **FD-22a** and **FD-24a** displayed a high degree of selectivity for CB2R relative to CB1R, whereas their constituent orthosteric ligand—**FM-6b**—did not display receptor subtype selectivity. This is particularly important for cannabinoid receptors given the longstanding issues associated with developing receptor subtype-selective agonists.^49^ Furthermore, **FD-22a** and **FD-24a** displayed a greater affinity to CB2R than **FM-6b**, although this difference was not significant, and **FM-6b** already displayed nanomolar affinity. Finally, **FD-22a** and **FD-24a** were more potent in the cAMP inhibition assay than the βarrestin2 recruitment assay. Although we did not directly estimate bias or compare the biases of bitopic ligands to **FM-6b**, G protein-selectivity does appear to have been enhanced through the use of these bitopic ligands.

Our compounds **FD-22a** and **FD-24a** were designed by linking the pharmacophoric portion of the CB2R PAM **EC-21a** to that of the CB2R orthosteric agonist **FM-6b** through an alkyl chain characterized by the presence of a 1,2,3-triazole ring. Binding and functional cAMP studies revealed that both compounds were able to selectively activate CB2R versus CB1R. Control experiments in CHO–K1 cells lacking either cannabinoid receptor indicate that additional targets are engaged by these compounds beyond the endogenous cannabinoid system, and this activity will be the subject of future studies. **FD-22a** and **FD-24a** only enhanced βarrestin2 recruitment at concentrations above 1 μM, whereas both compounds displayed nanomolar potency in the cAMP inhibition assay, highlighting a functional selectivity for the cAMP inhibition. Similarly, the parent agonist **FM-6b** displayed a greater potency and efficacy in the cAMP inhibition assay relative to the βarrestin2 recruitment assay, suggesting potential functional selectivity for the bitopic ligands was derived from **FM-6b.** Additional studies based on the co-administration of **FD-22a** or **FD-24a** with the CB2R PAM **EC-21a** or with the CB2R antagonist/inverse agonist SR144528 showed that **FD-22a** and **FD-24a** were able to simultaneously bind to both CB2R orthosteric and allosteric binding sites, although **FD-24a** displayed a lower affinity for the allosteric site and a higher affinity for the orthosteric site as compared to **FD-22a**. Computational studies showed that these compounds appear to preserve the usual orthosteric binding mode and engage in interactions with a unconserved region of the CB2R surface, which is suggested as a potential allosteric binding site close to residue Ser193. The correlation between the linker length and the ability to inhibit FSK-stimulated cAMP accumulation was rationalized by docking, suggesting the role of longer linkers for occupying both orthosteric and allosteric site and of the triazole as a stabilizer of the aromatic residues Tyr190 and Trp194. Additional studies are required to understand the potential bitopic pharmacology of **FD-22a** and **FD-24a**. Based on prior studies of bitopic ligands at other GPCRs,^14,60,61^ mutagenesis studies assessing compound sensitivity to the orthosteric site and allosteric site mutations, interaction studies with other orthosteric ligands to assess probe dependence, and estimation of ligand bias would provide critical insights into how **FD-22a** and **FD-24a** function as CB2R bitopic ligands.

Subsequently, both compounds were tested to evaluate their ability to decrease the inflammatory phenotype of LPS-stimulated BV2 microglial cells, and **FD-22a** was found to be the most potent in preventing the inflammatory response induced by an LPS stimulus. Notably, as demonstrated by pharmacological antagonism, the observed anti-inflammatory effects were revealed to be CB2R-mediated. **FD-22a** was also tested in a human microglial cell (HMC3) inflammatory model. The results confirmed that **FD-22a** used at 1 μM concentration was capable of producing significant CB2R-mediated anti-inflammatory effects. Finally, **FD-22a** did not show any relevant cytotoxic effects in microglial cells, as assessed using the MTT reduction assay. In conclusion, our results showed that the newly developed CB2R heterobivalent bitopic ligand, **FD-22a,** significantly contrasts the inflammatory process in microglial cells, counteracting a mechanism that supports the onset, progression, and severe symptomatology of several neurodegenerative diseases, including Alzheimer’s disease^62^ Parkinson’s disease,^63^ and amyotrophic lateral sclerosis^64^ and psychiatric disorders.^65^

The anti-inflammatory action in microglia cells mimicking inflammatory conditions has been already reported for several CB2R agonists.^66^ However, the molecular mechanisms that underlie the success of these treatments have not been clearly defined,^67^ and there is still a need for a clear understanding of CB2R signaling in activated microglia.

Despite the promising potential of CB2R agonists,^68^ their main limitations are common side effects mainly related to the internalization of CB2R. Indeed, it has been reported that CB2R agonists, by enhancing βarrestin2 recruitment, can induce internalization and desensitization of the receptor, leading to a decrease in signaling and surface receptor levels.^69^ Our bitopic ligand **FD-22a** showed no enhancement of βarrestin2 recruitment, thereby presenting biased properties that could induce beneficial effects of neuroprotection with fewer side effects.^70^

The bitopic ligand **FD-22a** was also tested in cold allodynia assays to investigate its effect on neuropathic pain. Results showed that **FD-22a**, after oral administration, dose-dependently reversed the lowering of the threshold to cold stimuli (cold plate test) induced by oxaliplatin. As demonstrated by pharmacological antagonism, this effect is mediated by CB2R. Several cell types, including microglia, are involved in the maladaptive plasticity of the nervous tissue that is thought to be the basis of chronic pain,^71^ so **FD-22a** could beneficially influence CNS damages induced by neuropathies.

The design of dualsteric/bitopic agents represents a novel strategy in medicinal chemistry. Dualsteric/bitopic ligands can open the door for selective drug effects and can be considered valuable tools for a better understanding of the receptor activation process. In conclusion, **FD-22a** can be useful for a better understanding of the physiological effects related to the bitopic stimulation of CB2R, which can lead to numerous beneficial therapeutic applications. CB2R activation has been recently shown to produce several neuroprotective effects,^72^ and bitopic ligands of CB2R, engaging simultaneously the orthosteric and allosteric sites, could be useful in neurodegenerative disease when the endogenous tone is progressively reduced.

## Experimental Section

### Chemistry

Commercially available reagents were purchased from Sigma-Aldrich, Tokyo Chemical Industry or Fluorochem and used without purification. ^1^H NMR and ^13^C NMR were recorded at 400 and 100 MHz, respectively, on a Bruker AVANCE IIITM 400 spectrometer. The chemical shift (δ) is reported in parts per million related to the residual solvent signal, while coupling constants (J) are expressed in Hertz (Hz). All compounds are >95% pure by HPLC analysis. The analytical HPLC system consisted of a Varian 9012 solvent delivery system coupled to a Varian ProStar 330 DAD detector with operating wavelengths in the range between 200 and 400 nm, and Star LC Workstation version 6.41 software was used for instrument control, data acquisition, and data processing. Analyses were performed on a reverse phase C18 column [Luna C18(2) 150 mm × 4.6 mm, 5 μm particle size, Phenomenex]. The mobile phase was constituted by H_2_O (eluent A) and ACN (eluent B) at a flow rate of 600 μL/min. A linear gradient starting from 40% of A, changing to 80% of A over 30 min, and returning to the initial conditions over 20 min was used. The target compound is ≥ 98% pure by HPLC analysis (Supporting Information).

High-resolution mass spectra (HRMS) were recorded on a Q Exactive Plus Hybrid Quadrupole-Orbitrap mass spectrometer (Thermo Fisher Scientific), equipped with an HESI source. The ESI-MS spectrum was recorded by direct injection at a 5 μ mL min^–1^ flow rate. Working conditions: positive polarity, spray voltage 3.5 kV, capillary temperature 300 °C, S-lens RF level 55, sheath gas 20, auxiliary gas 3 (arbitrary units); negative polarity, spray voltage 3.4 kV, capillary temperature 270 °C, S-lens RF level 55, sheath gas 35, auxiliary gas 8 (arbitrary units). Acquisition and analysis: Xcalibur 4.2 software (Thermo). For spectra acquisition a nominal resolution (at *m*/*z* 200) of 140,000 was used. Organic solutions were dried over anhydrous Na_2_SO_4_. Evaporation was carried out in vacuo using a rotating evaporator. Silica gel flash chromatography was performed using silica gel 60 Å (0.040–0.063 mm; Merck Life Science S.r.l.). Reactions were monitored by TLC on Merck aluminium silica gel (60 F254) plates that were visualized under a UV lamp (λ = 254 nm). Melting points were determined on a Kofler hot-stage apparatus and are uncorrected.

#### Methyl 1,2-Dihydro-6-methyl-2-*oxo*-pyridine-3-carboxylate (**1**)

To a solution of commercially available 6-methyl-2-*oxo*-1,2-dihydropyridine-3-carboxylic acid (2.00 g, 13.06 mmol) in methanol (30 mL), H_2_SO_4_ 96% was added. The reaction mixture was stirred at 90 °C for 24 h. The solution was treated with NaHCO_3_, until pH 7–8 was reached and then extracted with dichloromethane. The organic phase was dried over anhydrous Na_2_SO_4_, filtered, and evaporated under reduced pressure to afford compound **1** (1.58 g, 9.45 mmol) as a white solid. Yield: 72%. ^1^H NMR: (CDCl_3_) δ (ppm) 11.03 (bs, 1H, NH), 8.18 (d, 1H, *J* = 7.4 Hz, H_4_-Py), 6.27 (d, 1H, *J* = 7.4 Hz, H_5_-Py), 3.90 (s, 3H, −OCH_3_), 2.46 (s, 3H, CH_3_-Py).

#### *N*-Cycloheptyl-1,2-Dihydro-6-methyl-2-*oxo*-pyridine-3-carboxamide (**2**)

A mixture of compound **1** (1.64 g, 9.81 mmol) and cycloheptylamine (8.21 mL) was heated in a sealed tube at 100 °C for 24 h. After cooling at 0 °C, the reaction mixture was treated with 10% aqueous HCl until pH = 4. The solid precipitate was collected by filtration, affording compound **2** (1.85 g, 7.45 mmol) as a white solid. Yield: 76%. ^1^H NMR: (CDCl_3_) δ (ppm) 12.95 (bs, 1H, OH), 9.63 (bd, 1H, *J* = 7.6 Hz, NH), 8.52 (d, 1H, *J* = 7.4 Hz, H_4_-Py), 6.34 (d, 1H, *J* = 7.4 Hz, H_5_-Py), 4.20 (m, 1H, CHN), 2.44 (s, 3H, CH_3_-Py), 1.97 (m, 2H, CH_2_), 1.61 (m, 10H, 5 × CH_2_).

#### *N*-Cycloheptyl 1,2-Dihydro-5-bromo-6-methyl-2-*oxo*-pyridine-3-carboxamide (**3**)

Compound **2** (0.606 g, 2.44 mmol) was dissolved in CHCl_3_, and a solution of bromine (0.31 mL, 6.11 mmol) in CHCl_3_ (4.1 mL) was added dropwise. The reaction mixture was stirred at room temperature for 12 h and then diluted with CHCl_3_. The solution was treated with a saturated solution of sodium thiosulfate and then washed with water. The organic phase was dried over anhydrous Na_2_SO_4_, filtered, and evaporated under reduced pressure to give compound **3** (0.792 mg, 2.42 mmol) as a yellow solid, which was used in the next step without further purification. Yield: 99%. ^1^H NMR: (CDCl_3_) δ (ppm) 12.98 (bs, 1H, OH), 9.39 (bd, 1H, *J* = 7.6 Hz NH), 8.65 (s, 1H, H_4_-Py), 4.18 (m, 1H, CHN), 2.58 (s, 3H, CH_3_-Py), 1.98 (m, 2H, CH_2_), 1.60 (m, 10H, 5 × CH_2_).

#### 5-Bromo-1-(5-bromopentyl)-*N*-cycloheptyl-1,2-Dihydro-6-methyl-2-*oxo*-pyridine-3-carboxamide (**4**) and 5-Bromo-2-((5-bromopentyl)oxy)-*N*-cycloheptyl-6-methylnicotinamide (**6**)

Cesium fluoride (0.975 g, 6.42 mmol) was added to a solution of compound **3** (0.700 g, 2.14 mmol) in anhydrous DMF (6.50 mL). After 1 h, 1,5-dibromopentane (0.87 mL, 6.42 mmol) was added, and the resulting mixture was left under stirring at 30 °C for 12 h. DMF was removed under reduced pressure. The residue obtained was dissolved in CHCl_3_ and washed three times with water. The organic phase was dried over Na_2_SO_4_, filtered, and evaporated under reduced pressure. The crude product obtained was purified by flash chromatography on silica gel using ethyl acetate/hexane 3:7 as an eluent to afford compounds **4** (0.110 g, 0.230 mmol) and **6** (0.370 g, 0.780 mmol). **4**: Yield: 11%. ^1^H NMR: (CDCl_3_) δ (ppm) 9.64 (bd, 1H, *J* = 7.2 Hz, NH), 8.56 (s, 1H, H_4_-Py), 4.12 (m, 3H, CHN + CH_2_NCO), 3.43 (t, 2H, *J* = 6.2 Hz, CH_2_–Br), 2.62 (s, 3H, CH_3_-Py), 1.94 (m, 4H, 2 × CH_2_), 1.66 (m, 14H, 7 × CH_2_). **6**: Yield: 36%. ^1^H NMR: (CDCl_3_) δ (ppm) 8.54 (s, 1H, H_4_-Py), 7.89 (bd, 1H, *J* = 7.8 Hz, NH), 4.47 (t, 2H, *J* = 6.2 Hz, CH_2_O-Py), 4.15 (m, 1H, CHN), 3.44 (t, 2H, *J* = 6.6 Hz, CH_2_–Br), 2.56 (s, 3H, CH_3_-Py), 1.93 (m, 6H, 3 × CH_2_), 1.66 (m, 12H, 6 × CH_2_).

#### 5-Bromo-1-(3-bromopropyl)-*N*-cycloheptyl-1,2-Dihydro-6-methyl-2-*oxo*-pyridine-3-carboxamide (**5**) and 5-Bromo-2-(3-bromopropoxy)-*N*-cycloheptyl-6-methylnicotinamide (**7**)

Compounds **5** and **7** were prepared from compound **3**, as described for compounds **4** and **6** using 1,3-dibromopropane and purified by flash column chromatography on silica gel using ethyl acetate/hexane 3:7 as an eluent. **5**: Yield: 37%. ^1^H NMR: (CDCl_3_) δ (ppm) 9.57 (bd, 1H, *J* = 7.6 Hz, NH), 8.58 (s, 1H, H_4_-Py), 4.31 (m, 2H, CH_2_NCO), 4.13 (m, 1H, CHN), 3.53 (t, 2H, *J* = 6.0 Hz, CH_2_–Br), 2.67 (s, 3H, CH_3_-Py), 2.28 (m, 2H, CH_2_), 1.98 (m, 2H, CH_2_), 1.63 (m, 10H, 5 × CH_2_). **7**: Yield: 12%. ^1^H NMR: (CDCl_3_) δ (ppm) 8.54 (s, 1H, H_4_-Py), 7.76 (bd, 1H, *J* = 7.6 Hz, NH), 4.65 (t, 2H, *J* = 6.0 Hz, CH_2_O-Py), 4.16 (m, 1H, CHN), 3.53 (t, 2H, *J* = 6.6 Hz, CH_2_–Br), 2.56 (s, 3H, CH_3_-Py), 2.37 (m, 2H, CH_2_), 2.00 (m, 2H, CH_2_), 1.59 (m, 10H, 5 × CH_2_).

#### 1-(5-Azidopentyl)-5-bromo-*N*-cycloheptyl-1,2-Dihydro-6-methyl-2-*oxo*-pyridine-3-carboxamide (**8**)

Compound **4** (0.110 g, 0.230 mmol) was dissolved in anhydrous DMF, in a vial. Then, NaN_3_ (0.045 g, 0.690 mmol) was added. The reaction mixture was stirred at 60 °C for 12 h. DMF was removed under reduced pressure. The residue was dissolved in CHCl_3_ and washed three times with water. The organic phase was dried over Na_2_SO_4_, filtered, and evaporated under reduced pressure, to afford compound **8** (0.070 g, 0.160 mmol) as yellow oil. Yield: 70%. ^1^H NMR: (CDCl_3_) δ (ppm) 9.65 (bd, 1H, *J* = 7.6 Hz, NH), 8.57 (s, 1H, H_4_-Py), 4.14 (m, 3H, CHN + CH_2_NCO), 3.33 (t, 2H, *J* = 6.6 Hz, CH_2_–N_3_), 2.63 (s, 3H, CH_3_-Py), 1.98 (m, 2H, CH_2_), 1.63 (m, 16H, 8 × CH_2_).

#### 2-((5-Azidopentyl)oxy)-5-bromo-*N*-cycloheptyl-6-methylnicotinamide (**10**)

Compound **10** was prepared from compound **6** as described for compound **8**. Yield: 85%. ^1^H NMR: (CDCl_3_) δ (ppm) 8.54 (s, 1H, H_4_-Py), 7.89 (bd, 1H, *J* = 7.8 Hz, NH), 4.46 (t, 2H, *J* = 6.2 Hz, CH_2_O-Py), 4.15 (m, 1H, CHN), 3.32 (t, 2H, *J* = 6.6 Hz, CH_2_–N_3_), 2.56 (s, 3H, CH_3_-Py), 2.00 (m, 2H, CH_2_), 1.86 (m, 2H, CH_2_), 1.64 (m, 14H, 7 × CH_2_).

#### 1-(3-Azidopropyl)-5-bromo-*N*-cycloheptyl-1,2-Dihydro-6-methyl-2-*oxo*-pyridine-3-carboxamide (**9**)

Compound **9** was prepared from compound **5** as described for compound **8**. Yield: 69%. ^1^H NMR: (CDCl_3_) δ (ppm) 9.60 (bd, 1H, *J* = 7.2 Hz, NH), 8.58 (s, 1H, H_4_-Py), 4.24 (t, 2H, *J* = 7.6 Hz, CH_2_NCO), 4.13 (m, 1H, CHN), 3.49 (t, 2H, *J* = 6.2 Hz, CH_2_–N_3_), 2.64 (s, 3H, CH_3_-Py), 1.98 (m, 4H, 2 × CH_2_), 1.60 (m, 10H, 5 × CH_2_).

#### 2-(3-Azidopropoxy)-5-bromo-*N*-cycloheptyl-6-methylnicotinamide (**11**)

Compound **11** was prepared from compound **7** as described for compound **8**. Yield: 68%. ^1^H NMR: (CDCl_3_) δ (ppm) 8.54 (s, 1H, H_4_-Py), 7.79 (bd, 1H, *J* = 7.6 Hz, NH), 4.57 (t, 2H, *J* = 6.2 Hz, CH_2_O-Py), 4.15 (m, 1H, CHN), 3.51 (t, 2H, *J* = 6.6 Hz, CH_2_–N_3_), 2.56 (s, 3H, CH_3_-Py), 2.10 (m, 2H, CH_2_), 2.01 (m, 2H, CH_2_), 1.64 (m, 10H, 5 × CH_2_).

#### 3-Amino-4-methylpyridin-**2**(**1*H***)-one (**12**)

Iron powder (1.825 g, 32.63 mmol) and ammonium chloride (0.922 g, 17.23 mmol) were added to a solution of the commercially available 2-hydroxy-4-methyl-3-nitropyridine (0.400 g, 2.61 mmol) in ethanol (16 mL) and water (8 mL). The reaction mixture was refluxed for 3 h, filtered under vacuum using Celite, and evaporated under reduced pressure. The obtained residue was dissolved in CHCl_3_ and washed with water. The organic phase was dried over Na_2_SO_4_, filtered, and evaporated under reduced pressure giving the desired compound **12** as a brown solid (0.294 g, 2.37 mmol), which was used in the next step without any further purification. Yield: 91%. ^1^H NMR (CDCl_3_) δ: 12.27 (bs, 1H, NH), 6.77 (d, 1H, J = 6.8 Hz, H_6_-Py), 6.07 (d, 1H, J = 6.8 Hz, H_5_-Py), 4.01 (bs, 2H, NH_2_), 2.09 (s, 3H, CH_3_-Py).

#### *N*-(1,2-Dihydro-4-methyl-2-oxopyridin-3-yl)-Cycloheptanecarboxamide (**13**)

Cycloheptane carboxylic acid (0.86 mL, 6.29 mmol) was dissolved in C_2_O_2_Cl_2_ (1.59 mL, 18.87 mmol) with 3 drops of DMF. The solution was stirred at room temperature for 0.5 h, and then the excess of C_2_O_2_Cl_2_ was removed by evaporation under nitrogen flux. The obtained acyl chloride was added dropwise to a solution of **12** (0.585 g, 4.71 mmol) and triethylamine (3.18 mL, 23.56 mmol) in anhydrous DMF at 0 °C. The reaction mixture was stirred at room temperature for 24 h, and then the solvent was removed under reduced pressure to give a residue which was dissolved in CHCl_3_ and washed three times with water. Subsequently, the organic phase was dried over Na_2_SO_4_, filtered, and evaporated under reduced pressure to afford a brown oil, which was triturated in methanol to obtain compound **13** as a white solid (0.621 g, 2.50 mmol). Yield: 53%. ^1^H NMR (CDCl_3_) δ: 12.26 (bs, 1H, NH), 7.51 (bs, 1H, NH), 7.14 (d, 1H, J = 6.8 Hz, H_6_-Py), 6.27 (d, 1H, J = 6.8 Hz, H_5_-Py), 2.53 (m, 1H, CHCO), 2.18 (s, 3H, CH_3_-Py), 2.02 (m, 2H, CH_2_), 1.78 (m, 4H, 2 × CH_2_), 1.58 (m, 6H, 3 × CH_2_).

#### *N*-(5-bromo-1,2-Dihydro-4-methyl-2-oxopyridin-3-yl)-Cycloheptanecarboxamide (**14**)

A solution of Br_2_ (0.14 mL, 2.73 mmol) in CHCl_3_ (1.81 mL) was added dropwise to a solution of derivative **8** (0.270 g, 1.09 mmol) in CHCl_3_. The reaction mixture was stirred at room temperature overnight and then was washed four times with a saturated aqueous solution of Na_2_S_2_O_3_. The organic phase was then dried over Na_2_SO_4_, filtered, and evaporated under reduced pressure to afford a soid residue, which was triturated in ethyl acetate, giving the desired compound **14** (0.330 g, 1.00 mmol) as a beige solid. Yield: 92%. ^1^H NMR (CDCl_3_) δ: 12.23 (bs, 1H, NH), 7.50 (bs, 1H, NH), 7.43 (s, 1H, H_6_-Py), 2.53 (m, 1H, CHCO), 2.23 (s, 3H, CH_3_-Py), 2.02 (m, 2H, CH_2_), 1.78 (m, 4H, 2 × CH_2_), 1.54 (m, 6H, 3 × CH_2_).

#### *N*-(5-bromo-1,2-Dihydro-4-methyl-2-*oxo*-1-(pent-4-yn-1-yl)-pyridin-3yl)-Cycloheptanecarboxamide (**15**)

Cesium fluoride (0.638 g, 4.20 mmol) was added to a solution of compound **14** (0.458 g, 1.40 mmol) in anhydrous DMF (4.20 mL). After 1 h at room temperature, 5-chloro-1-pentyne (0.44 mL, 4.20 mmol) was added, and the resulting mixture was left under stirring at 30 °C for 12 h. After that the solvent was removed under reduced pressure, and the residue obtained was dissolved in CHCl_3_ and washed three times with water. The organic phase was dried over Na_2_SO_4_, filtered, and evaporated under reduced pressure yielding a crude product which was purified by flash chromatography on silica gel using ethyl acetate/hexane 4:6 as eluent to afford compound **15** (0.416 g, 1.06 mmol) as a white solid. Yield: 76%. mp: 117–120 °C. ^1^H NMR: (CDCl_3_) δ (ppm) 7.54 (bs, 1H, NH), 7.38 (s, 1H, *H*_6_-Py), 4.03 (t, 2H, *J* = 7.0 Hz, CH_2_NCO), 2.50 (m, 1H, CHCO), 2.25 (dt, 2H, *J* = 6.8 Hz, *J* = 2.6 Hz, CH_2_C), 2.15 (s, 3H, CH_3_-Py), 2.05 (t, 1H, *J* = 2.6 Hz, CCH), 1.98 (m, 4H, 2 × CH_2_), 1.75 (m, 4H, 2 × CH_2_), 1.54 (m, 6H, 3 × CH_2_).

#### *N*-(5-bromo-1,2-Dihydro-4-methyl-2-*oxo*-1-(prop-2-yn-1-yl)-pyridin-3-yl)-Cycloheptanecarboxamide (**16**)

Compound **16** was prepared from compound **14**, as described for compound **15**, using propargyl bromide and purified by flash column chromatography on silica gel using ethyl acetate/hexane 4:6 as an eluent. Yield: 54%. mp: 175–178 °C. ^1^H NMR: (CDCl_3_) δ (ppm) 7.65 (bs, 1H, NH), 7.42 (s, 1H, H_6_-Py), 4.72 (d, 2H, J = 2.6 Hz, CH_2_NCO), 2.52 (t, 1H, J = 2.6 Hz, CCH), 2.50 (m, 1H, CHCO), 2.17 (s, 3H, CH_3_-Py), 1.99 (m, 2H, CH_2_), 1.77 (m, 4H, 2 × CH_2_), 1.56 (m, 6H, 3 × CH_2_).

#### 5-Bromo-1-(5-(4-(3-(5-bromo-3-(Cycloheptanecarboxamido)-4-methyl-2-oxopyridin-1(2*H*)-yl) propyl)-1*H*-1,2,3–Triazol–1-yl)pentyl)–*N*–Cycloheptyl–6–methyl–2–*oxo*-1,2-Dihydropyridine–3-Carboxamide (**FD-22a**)

To a solution of compounds **8** (0.044 g, 0.10 mmol) and **15** (0.039 g, 0.10 mmol) in DMF (2.45 mL) and water (0.61 mL), CuSO_4_·5H_2_O (0.025 g, 0.10 mmol) and sodium ascorbate (0.057 g, 0.29 mmol) were added. The reaction mixture was stirred at 80 °C for 2 h. Subsequently, the solvent was removed under reduced pressure to give a residue, which was dissolved in ethyl acetate and washed with saturated solution of NaHCO_3_. The organic phase was dried over Na_2_SO_4_, filtered, and evaporated under reduced pressure. The crude product obtained was purified by flash chromatography on silica gel using chloroform and 2% of methanol as an eluent to afford compound **FD-22a** (0.042 mg, 0.05 mmol) as an oil. Yield: 52%. ^1^H NMR: (CDCl_3_) δ (ppm) 9.61 (bd, 1H, *J* = 8.0 Hz, NH), 8.56 (s, 1H, H_4_-Py), 7.47 (bs, 1H, NH), 7.43 (s, 1H, H_6_-Py), 7.35 (s, 1H, NCHC), 4.37 (t, 2H, *J* = 6.8 Hz, CH_2_-triazole), 4.10 (m, 3H, CH_2_NCO + CHNH), 4.01 (t, 2H, *J* = 6.8 Hz, CH_2_NCO), 2.77 (t, 2H, *J* = 7.0 Hz, CH_2_-triazole), 2.60 (s, 3H, CH_3_), 2.51 (m, 1H, CHCO), 2.14 (m, 5H, CH_3_ + CH_2_), 2.00 (m, 6H, CH_2_ × 3), 1.66 (m, 24H, CH_2_ × 12). ^13^C NMR: (CDCl_3_) δ (ppm) 176.16 (C=O), 161.68, 161.66 (2 × C=O), 158.45 (C_2_-Py), 147.72 (C_6_-Py), 146.02 (C_4_-Py), 142.18 (C_3_-Py), 133.07 (C_4_-Py), 126.36 (C_6_-Py), 121.09 (*C*-triazole), 119.77 (C_3_-Py), 115.40 (*C*-triazole), 103.60 (C_5_-Py), 101.77 (C_5_-Py), 50.56 (CHN), 49.89 (CH_2_-triazole), 49.41 (CH_2_-Py), 47.85 (CHCO), 46.54 (CH_2_-Py), 35.01 (2 × CH_2_CHN), 31.81 (2 × CH_2_CHCO), 29.84 (CH_2_-triazole), 28.51 (CH_2_), 28.23 (2 × CH_2_), 28.20 (2 × CH_2_), 27.61 (CH_2_), 26.69 (2 × CH_2_), 24.26 (2 × CH_2_), 23.90 (CH_2_), 22.52 (CH_2_), 20.69 (CH_3_-Py), 20.48 (CH_3_-Py). HRMS-ESI: *m/z* calcd for C_38_H_53_N_7_O_4_Br_2_ [M–H]^−^, 828.24530; found, 828.24713.

#### 5-Bromo-1-(3-(4-(3-(5-bromo-3-(Cycloheptanecarboxamido)-4-methyl-2-oxopyridin-1(2*H*)-yl)propyl)-1*H*-1,2,3-triazol-1-yl)propyl)-*N*-cycloheptyl-6-methyl-2-*oxo*-1,2-dihydropyridine-3-carboxamide (**FD-25a**)

Compound **FD-25a** was prepared from compounds **9** and **15**, as described for compound **FD-22a** and purified by flash column chromatography using ethyl acetate and methanol 2% as an eluent. Yield: 77%. ^1^H NMR: (CDCl_3_) δ (ppm) 9.56 (bd, 1H, *J* = 8.0 Hz, NH), 8.57 (s, 1H, H_4_-Py), 7.43 (s, 1H, NH), 7.40 (s, 1H, H_6_-Py), 7.29 (s, 1H, NCHC), 4.46 (t, 2H, *J* = 6.8 Hz, CH_2_-triazole), 4.24 (t, 2H, *J* = 7.8 Hz, CH_2_NCO), 4.12 (m, 1H, CHNH), 4.01 (t, 2H, *J* = 7.0 Hz, CH_2_NCO), 2.77 (t, 2H, *J* = 7.0 Hz, CH_2_- triazole), 2.52 (s, 3H, CH_3_), 2.50 (m, 1H, CHCO), 2.36 (m, 2H, CH_2_), 2.17 (s, 3H, CH_3_), 2.15 (m, 2H, CH_2_), 1.99 (m, 4H, 2 × CH_2_), 1.77 (m, 4H, 2 × CH_2_), 1.57 (m, 16H, 8 × CH_2_). ^13^C NMR: (CDCl_3_) δ (ppm) 176.12 (C=O), 161.78, 161.46 (2 × C=O), 158.41 (C_2_-Py), 147.85 (C_6_-Py), 146.25 (C_4_-Py), 142.37 (C_3_-Py), 133.01 (C_4_-Py), 126.36 (C_6_-Py), 121.64 (*C*-triazole), 119.75 (C_3_-Py), 115.30 (*C*-triazole), 103.49 (C_5_-Py), 101.95 (C_5_-Py), 50.48 (CHN), 49.28 (CH_2_-triazole), 47.82 (CH_2_-Py), 47.78 (CHCO), 44.30 (CH_2_-Py), 34.93 (2 × CH_2_CHN), 31.78 (2 × CH_2_CHCO), 28.50 (CH_2_-triazole), 28.40 (CH_2_), 28.20 (2 × CH_2_), 28.16 (2 × CH_2_), 26.65 (2 × CH_2_), 24.20 (2 × CH_2_), 22.47 (CH_2_), 20.52 (CH_3_-Py), 20.39 (CH_3_-Py). HRMS-ESI: *m/z* calcd for C_36_H_49_N_7_O_4_Br_2_ [M–H]^−^, 800.21400; found, 800.21564.

#### 5-Bromo-1-(5-(4-((5-bromo-3-(cycloheptanecarboxamido)-4-methyl-2-oxopyridin-1(2*H*)-yl)methyl)-1*H*-1,2,3-triazol-1-yl)pentyl)-*N*-cycloheptyl-6-methyl-2-*oxo*-1,2-Dihydropyridine-3-carboxamide (**FD-27a**)

Compound **FD-27a** was prepared from compounds **8** and **16,** as described for compound **FD-22a** and purified by flash column chromatography using ethyl acetate and methanol 2% as an eluent. Yield: 27%. ^1^H NMR: (CDCl_3_) δ (ppm) 9.61 (bd, 1H, *J* = 8.0 Hz, NH), 8.55 (s, 1H, H_4_-Py), 7.67 (s, 2H, NH + H_6_-Py), 7.43 (s, 1H, NCHC), 5.17 (s, 2H, CCH_2_N), 4.35 (t, 2H, *J* = 7.0 Hz, CH_2_-triazole), 4.12 (m, 3H, CH_2_NCO + CHNH), 2.59 (s, 3H, CH_3_), 2.49 (m, 1H, CHCO), 2.14 (s, 3H, CH_3_), 1.98 (m, 6H, CH_2_ × 3), 1.68 (m, 24H, CH_2_ × 12). ^13^C NMR: (CDCl_3_) δ (ppm) 175.94 (C=O), 161.53 (C=O + C_2_-Py), 158.15 (C_2_-Py), 147.53 (C_6_-Py), 145.87 (C_4_-Py), 142.92 (C_3_-Py), 132.78 (C_4_-Py), 126.05 (C_6_-Py), 120.09 (*C*-triazole), 119.69 (C_3_-Py), 115.12 (*C*-triazole), 103.80 (C_5_-Py), 101.62 (C_5_-Py), 50.44 (CHN), 50.01 (CH_2_-triazole), 47.73 (CHCO), 46.36 (CH_2_-Py), 44.38 (CH_2_-Py), 34.88 (2 × CH_2_CHN), 31.68 (2 × CH_2_CHCO), 29.58 (CH_2_), 28.07 (4 × CH_2_), 27.46 (CH_2_), 26.55 (2 × CH_2_), 24.14 (2 × CH_2_), 23.80 (CH_2_), 20.54 (CH_3_-Py), 20.33 (CH_3_-Py). HRMS-ESI: *m/z* calcd for C_36_H_49_N_7_O_4_Br_2_ [M–H]^−^, 800.21400; found, 800.21625.

#### 5-Bromo-1-(3-(4-((5-bromo-3-(cycloheptanecarboxamido)-4-methyl-2-oxopyridin-1(2*H*)-yl)methyl)-1*H*-1,2,3-triazol-1-yl)propyl)-*N*-cycloheptyl-6-methyl-2-*oxo*-1,2-Dihydropyridine-3-carboxamide (**FD-32a**)

Compound **FD-32a** was prepared from compounds **9** and **16**, as described for compound **FD-22a** and purified by flash column chromatography using ethyl acetate and methanol 2% as an eluent. Yield: 47%. ^1^H NMR: (CDCl_3_) δ (ppm) 9.57 (bd, 1H, *J* = 8.0 Hz, NH), 8.49 (s, 1H, H_4_-Py), 7.71 (bs, 1H, NH), 7.65 (s, 1H, H_6_-Py), 7.53 (s, 1H, NCHC), 5.14 (s, 2H, CCH_2_N), 4.47 (t, 2H, *J* = 6.8 Hz, CH_2_-triazole), 4.26 (t, 2H, *J* = 7.4 Hz, CH_2_NCO), 4.12 (m, 1H, CHNH), 2.54 (m, 1H, CHCO), 2.50 (s, 3H, CH_3_-Py), 2.40 (m, 2H, 2 × CH_2_), 2.15 (s, 3H, CH_3_-Py), 2.00 (m, 4H, 2 × CH_2_), 1.77 (m, 4H, 2 × CH_2_), 1.59 (m, 16H, 8 × CH_2_). ^13^C NMR: (CDCl_3_) δ (ppm) 176.14 (C=O), 161.87 (C=O), 161.50 (C_2_-Py), 158.20 (C_2_-Py), 147.77 (C_6_-Py), 146.23 (C_4_-Py), 143.37 (*C*-triazole), 142.27 (C_3_-Py), 132.98 (C_4_-Py), 126.24 (C_6_-Py), 124.28 (*C*-triazole), 119.76 (C_3_-Py), 103.92 (C_5_-Py), 101.96 (C_5_-Py), 50.59 (CHN), 48.19 (CH_2_-triazole), 47.81 (CHCO), 44.55 (CH_2_-Py), 44.28 (CH_2_-Py), 34.97 (2 × CH_2_CHN), 31.81 (2 × CH_2_CHCO), 28.31 (CH_2_), 28.20 (2 × CH_2_), 28.19 (2 × CH_2_), 26.71 (2 × CH_2_), 24.25 (2 × CH_2_), 20.53 (CH_3_-Py), 20.40 (CH_3_-Py). HRMS-ESI: *m/z* calcd for C_34_H_45_N_7_O_4_Br_2_ [M–H]^−^, 772.18270; found, 772.18481.

#### 5-Bromo-2-((5-(4-(3-(5-bromo-3-(cycloheptanecarboxamido)-4-methyl-2-oxopyridin-1(2*H*)-yl)propyl)-1*H*-1,2,3-Triazol-1-yl)pentyloxy)-*N*-cycloheptyl-6-methylnicotinamide (**FD-24a**)

Compound **FD-24a** was prepared from compounds **10** and **15**, as described for compound **FD-22a** and purified by flash column chromatography using ethyl acetate and methanol 2% as an eluent. Yield: 31%. ^1^H NMR: (CDCl_3_) δ (ppm) 8.52 (s, 1H, H_4_-Py), 7.84 (bd, 1H, *J* = 8.0 Hz, NH), 7.48 (bs, 1H, NH), 7.42 (s, 1H, H_6_-Py), 7.34 (s, 1H, CH-triazole), 4.45 (t, 2H, *J* = 6.8 Hz, CH_2_O-Py), 4.36 (t, 2H, *J* = 7.2 Hz, CH_2_-triazole), 4.15 (m, 1H, CHNH), 4.01 (t, 2H, *J* = 7.2 Hz, CH_2_NCO), 2.76 (t, 2H, *J* = 7.0 Hz, CH_2_-triazole), 2.55 (s, 3H, CH_3_-Py), 2.50 (m, 1H, CHCO), 2.16 (s, 3H, CH_3_-Py), 2.13 (m, 2H, 2 × CH_2_), 2.00 (m, 4H, 2 × CH_2_), 1.84 (m, 2H, 2 × CH_2_), 1.76 (m, 6H, 3 × CH_2_), 1.56 (m, 18H, 9 × CH_2_). ^13^C NMR: (CDCl_3_) δ (ppm) 176.07 (C=O), 161.53 (C=O), 158.34 (C_2_-Py), 158.17 (C_2_-Py), 157.59 (C_6_-Py), 144.53 (C_4_-Py), 142.30 (C_3_-Py), 133.06 (C_4_-Py), 126.30 (C_6_-Py), 120.93 (*C*-triazole), 115.22 (C_3_-Py), 115.19 (*C*-triazole), 113.09 (C_5_-Py), 103.40 (C_5_-Py), 66.64 (CH_2_O-Py), 50.49 (CHN), 50.00 (CH_2_-triazole), 49.32 (CH_2_-Py), 47.72 (CHCO), 34.97 (2 × CH_2_CHN), 31.72 (2 × CH_2_CHCO), 29.99 (CH_2_-triazole), 28.41 (CH_2_), 28.16 (4 × CH_2_), 26.61 (2 × CH_2_), 24.53 (CH_2_), 24.06 (2 × CH_2_), 23.25 (CH_3_-Py), 22.44 (CH_2_), 20.36 (CH_3_-Py), 17.64 (CH_2_). HRMS-ESI: *m/z* calcd for C_38_H_53_N_7_O_4_Br_2_ [M–H]^−^, 828.24530; found, 828.24677.

#### 5-Bromo-2-(3-(4-(3-(5-bromo-3-(cycloheptanecarboxamido)-4-methyl-2-oxopyridin-1(2H)-yl)propyl)-1*H*-1,2,3-Triazol-1-yl)propoxy)-*N*-cycloheptyl-6-methylnicotinamide (**FD-30a**)

Compound **FD-30a** was prepared from compounds **11** and **15**, as described for compound **FD-22a** and purified by flash column chromatography using ethyl acetate and methanol 2% as an eluent. Yield: 78%. Mp: 168–170 °C. ^1^H NMR: (CDCl_3_) δ (ppm) 8.50 (s, 1H, H_4_-Py), 7.73 (bd, 1H, *J* = 8.0 Hz), 7.50 (s, 1H, NH), 7.40 (s, 1H, H_6_-Py), 7.29 (s, 1H, CH-triazole), 4.52 (t, 2H, *J* = 6.8 Hz, CH_2_O-Py), 4.47 (t, 2H, *J* = 6.8 Hz, CH_2_-triazole), 4.16 (m, 1H, CHN), 3.96 (t, 2H, *J* = 7.0 Hz, CH_2_N-Py), 2.76 (t, 2H, *J* = 6.8 Hz, CH_2_-triazole), 2.52 (s, 3H, CH_3_-Py), 2.50 (m, 1H, CHCO), 2.44 (m, 2H, CH_2_), 2.16 (s, 3H, CH_3_-Py), 2.12 (m, 2H, *C*–CH_2_–C), 2.03 (m, 4H, 2 × CH_2_), 1.79 (m, 4H, 2 × CH_2_), 1.62 (m, 16H, 8 × CH_2_). ^13^C NMR: (CDCl_3_) δ (ppm) 176.22 (C=O), 161.59 (C=O), 158.42 (C_2_-Py), 158.23 (C_2_-Py), 157.73 (C_6_-Py), 144.77 (C_4_-Py), 142.50 (C_3_-Py), 133.07 (C_4_-Py), 126.37 (C_6_-Py), 121.42 (*C*-triazole), 115.70 (C_3_-Py), 115.40 (*C*-triazole), 113.56 (C_5_-Py), 103.60 (C_5_-Py), 63.47 (CH_2_O-Py), 50.94 (CHN), 49.41 (CH_2_-triazole), 47.82 (CHCO), 46.98 (CH_2_-Py), 35.15 (2 × CH_2_CHN), 31.82 (2 × CH_2_CHCO), 29.75 (CH_2_-triazole), 28.30 (CH_2_), 28.24 (2 × CH_2_), 28.13 (2 × CH_2_), 26.72 (2 × CH_2_), 24.60 (CH_3_-Py), 24.27 (2 × CH_2_), 22.49 (CH_2_), 20.47 (CH_3_-Py). HRMS-ESI: *m/z* calcd for C_36_H_49_N_7_O_4_Br_2_ [M–H]^−^, 800.21400; found, 800.21558.

#### 5-Bromo-2-((5-(4-((5-bromo-3-(Cycloheptanecarboxamido)-4-methyl-2-oxopyridin-1(2*H*)-yl)methyl)-1*H*-1,2,3-Triazol-1-yl)pentyloxy)-*N*-cycloheptyl-6-methylnicotinamide (**FD-28a**)

Compound **FD-28a** was prepared from compounds **10** and **16**, as described for compound **FD-22a** and purified by flash column chromatography using ethyl acetate and methanol 2% as an eluent. Yield: 38% ^1^H NMR: (CDCl_3_) δ (ppm) 8.53 (s, 1H, H_4_-Py), 7.83 (bd, 1H, *J* = 8.0 Hz, NH), 7.67 (s, 1H, NH), 7.65 (s, 1H, H_6_-Py), 7.42 (s, 1H, CH-triazole), 5.16 (s, 2H, CH_2_N-Py), 4.46 (t, 2H, *J* = 6.8 Hz, CH_2_O-Py), 4.34 (t, 2H, *J* = 7.2 Hz, CH_2_-triazole), 4.12 (m, 1H, CHNH), 2.55 (s, 3H, CH_3_-Py), 2.50 (m, 1H, CHCO), 2.14 (s, 3H, CH_3_-Py), 1.99 (m, 6H, 3 × CH_2_), 1.85 (m, 2H, CH_2_), 1.77 (m, 4H, 2 × CH_2_), 1.57 (m, 18H, 9 × CH_2_). ^13^C NMR: (CDCl_3_) δ (ppm) 176.26 (C=O), 161.70 (C=O), 158.27 (C_2_-Py), 158.24 (C_2_-Py), 157.76 (C_6_-Py), 144.66 (C_4_-Py), 143.46 (*C*-triazole), 142.11 (C_3_-Py), 133.14 (C_4_-Py), 126.12 (C_6_-Py), 123.73 (*C*-triazole), 115.26 (C_3_-Py), 113.25 (C_5_-Py), 103.95 (C_5_-Py), 66.70 (CH_2_O-Py), 50.61 (CHN), 50.40 (CH_2_-triazole), 47.83 (CHCO), 44.51 (CH_2_-Py), 35.06 (2 × CH_2_CHN), 31.82 (2 × CH_2_CHCO), 30.00 (CH_2_), 28.48 (CH_2_), 28.25 (2 × CH_2_), 28.22 (2 × CH_2_), 26.71 (2 × CH_2_), 24.64 (CH_2_), 24.13 (2 × CH_2_), 23.40 (CH_3_-Py), 20.42 (CH_3_-Py). HRMS-ESI: *m/z* calcd for C_36_H_49_N_7_O_4_Br_2_ [M–H]^−^, 800.21400; found, 800.21576.

#### 5-Bromo-2-(3-(4-((5-bromo-3-(Cycloheptanecarboxamido)-4-methyl-2-oxopyridin-1(2*H*)-yl)methyl)-1*H*-1,2,3-Triazol-1-yl)propoxy)-*N*-cycloheptyl-6-methylnicotinamide (**FD-31a**)

Compound **FD-31a** was prepared from compounds **11** and **16**, as described for compound **FD-22a** and purified by flash column chromatography using ethyl acetate and methanol 2% as an eluent. Yield: 45%. ^1^H NMR: (CDCl_3_) δ (ppm) 8.53 (s, 1H, H_4_-Py), 7.71 (bd, 1H, *J* = 8.0 Hz), 7.69 (s, 1H, NH), 7.66 (s, 1H, H_6_-Py), 7.39 (s, 1H, CH-triazole), 5.16 (s, 2H, CH_2_NCO), 4.53 (t, 2H, *J* = 6.8 Hz, CH_2_O-Py), 4.49 (t, 2H, *J* = 6.8 Hz, CH_2_-triazole), 4.14 (m, 1H, CHNH), 2.56 (s, 3H, CH_3_-Py), 2.51 (m, 1H, CHCO), 2.44 (m, 2H, CH_2_), 2.14 (s, 3H, CH_3_-Py), 2.01 (m, 4H, 2 × CH_2_), 1.78 (m, 4H, 2 × CH_2_), 1.62 (m, 16H, 8 × CH_2_). ^13^C NMR: (CDCl_3_) δ (ppm) 176.30 (C=O), 161.58 (C=O), 158.27 (C_2_-Py), 157.73 (C_2_-Py), 157.64 (C_6_-Py), 144.77 (C_4_-Py), 143.65 (*C*-triazole), 142.24 (C_3_-Py), 133.15 (C_4_-Py), 126.07 (C_6_-Py), 124.00 (*C*-triazole), 115.56 (C_3_-Py), 113.59 (C_5_-Py), 103.98 (C_5_-Py), 63.55 (CH_2_O-Py), 50.94 (CHN), 47.75 (CH_2_-triazole), 47.40 (CHCO), 44.53 (CH_2_-Py), 35.06 (2 × CH_2_CHN), 31.78 (2 × CH_2_CHCO), 29.64 (CH_2_), 28.17 (2 × CH_2_), 28.08 (2 × CH_2_), 26.67 (2 × CH_2_), 24.59 (CH_3_-Py), 24.22 (2 × CH_2_), 20.38 (CH_3_-Py). HRMS-ESI: *m/z* calcd for C_34_H_45_N_7_O_4_Br_2_ [M–H]^−^, 772.18270; found, 772.18530.

### Biological Evaluation

#### Reagents and Cell Lines

CP55,940 was purchased from Cayman Chemicals (Ann Arbor, MI). [^3^H]CP55,940 (174.6 Ci/mmol) was obtained from PerkinElmer (Guelph, ON). LPS (*Escherichia coli* 0111:B4) and TNFα were purchased from Sigma-Aldrich (Milan, Italy), whereas SR144528 was from Tocris (Bristol, UK).

CHO–K1 cells untransfected or stably-expressing *h*CB1R or *h*CB2R were maintained, as described previously.^18,73^ Cells were maintained at 37 °C, 5% CO_2_ in F-12/DMEM containing 1 mM l-glutamine, 10% fetal bovine serum (FBS), and 1% Pen/Strep and hygromycin B (300 μg/mL) and G418 (600 μg/mL) for CHO–K1 *h*CB1R cells or G418 (400 μg/mL) for CHO–K1 *h*CB2R cells.^18,56^ In the case of membrane collection for radioligand binding, cells were scraped from flasks, centrifuged, and frozen at −80 °C until required. HitHunter (cAMP) and PathHunter (βarrestin2) CHO–K1 cells without additional receptor or stably-expressing *h*CB1R or *h*CB2R from DiscoveRx (Eurofins, Fremont, CA) were maintained at 37 °C, 5% CO_2_ in F-12 DMEM containing 10% FBS and 1% Pen/Strep with 800 μg/mL geneticin (HitHunter) or 800 μg/mL G418 and 300 μg/mL hygromycin B (PathHunter), as described previously.^18,73^

The BV-2 murine microglial cell line is an immortalized cell line with morphological, phenotypic, and functional properties associated with freshly isolated microglia, and thus, it is frequently used as an in vitro model to study microglial responses to pharmacological stimuli.^74,75^ BV-2 cells (CliniSciences, Guidonia Montecelio, Italy) were cultured in high-glucose DMEM (Corning, Tewksbury, MA, USA) supplemented with 10% FBS, streptomycin (100 g/mL), and penicillin (100 U/mL) (Sigma-Aldrich, Milan, Italy).

The human microglial clone 3 cell line (HMC3) (ATCC CRL-3304) consists of immortalized cells derived from human fetal brain that have become an ideal model for physiopathology research on neurodegenerative diseases, including Alzheimer’s disease, Parkinson’s disease, and dementia. HMC3 cells were cultured in high-glucose DMEM supplemented with 10% FBS, streptomycin (100 g/mL), and penicillin (100 U/mL) (Sigma-Aldrich, Milan, Italy).

#### HitHunter cAMP Assay

We have described quantification of FSK-stimulated cAMP accumulation using the DiscoveRx HitHunter assay elsewhere.^18,73^ To summarize, cells (20,000 cells/well in low-volume 96-well plates) were incubated overnight in Opti-MEM containing 1% FBS at 37 °C and 5% CO_2_. Opti-MEM media was then removed and replaced with cell assay buffer (DiscoveRx), and cells were cotreated at 37 °C with 10 μM FSK and ligands for 90 min. The cAMP antibody solution and cAMP working detection solutions were added to cells (DiscoveRx), and cells were incubated for 60 min at room temperature. cAMP solution A (DiscoveRx) was added, and cells were incubated for an additional 180 min at room temperature before chemiluminescence was measured on a Cytation5 plate reader (top read, gain 200, integration time 10,000 ms).

#### PathHunter CB1R βarrestin2 Assay

Similar to the cAMP inhibition assay, we have previously described the quantification of βarrestin2 recruitment using the DiscoveRx PathHunter assay.^18,73^ Briefly, cells (20,000 cells/well in low-volume 96-well plates) were incubated overnight in Opti-MEM containing 1% FBS at 37 °C and 5% CO_2_. Cells were treated with ligands for 90 min at 37 °C. A detection solution was added to cells (DiscoveRx), and cells were incubated for 60 min at room temperature. Chemiluminescence was measured on a Cytation5 plate reader (top read, gain 200, integration time 10,000 ms).

#### Radioligand Displacement Assay

These assays have been described in detail previously and are summarized here.^18,73^ Cells were thawed, diluted in Tris buffer (50 mM Tris–HCl and 50 mM Tris–base), and homogenized in a 1 mL handheld homogenizer.^18,73^*h*CB1R and *h*CB2R CHO–K1 cell membranes were collected by cavitation in a pressure cell and sedimented by ultracentrifugation. Pellets were resuspended in TME buffer (50 mM Tris–HCl, 5 mM MgCl_2_, 1 mM EDTA, pH 7.4), and protein concentration was measured *via* the Bradford method (Bio-Rad Laboratories, Mississauga, ON). Competition binding experiments were conducted with 1 nM [^3^H]CP55, 940 and Tris binding buffer (50 mM Tris–HCl, 50 mM Tris–base, 0.1% BSA, pH 7.4, 2 mL).^18,68^ Radioligand binding began with the addition of CHO–K1 cell membranes (50 μg protein per sample). Assays were performed for 120 min at 37 °C and stopped by the addition of ice-cold Tris binding buffer, followed by vacuum filtration using a 24-well sampling manifold (Brandel Cell Harvester; Brandel Inc, Gaithersburg, MD, USA). Brandel GF/B filter paper was soaked with wash buffer at 4 °C for at least 24 h. Each filter paper was washed 6 times with 1.2 mL aliquots of Tris-binding buffer then air-dried overnight and submerged in 5 mL of scintillation fluid (Ultima Gold XR, PerkinElmer). Liquid scintillation spectrometry was used to quantify radioactivity. For competition binding experiments, specific binding was equal to the difference in radioactivity with or without 1 μM unlabelled CP55, 940.

#### Analysis of Interleukin Release in Microglial Cell Inflammatory Models

The concentrations of proinflammatory and anti-inflammatory interleukins, namely IL-6 and IL-10, respectively, were determined by performing specific ELISA assays (MyBioSource, San Diego, CA, USA) on collected culture media. Murine microglial cells (BV2) were treated with test compounds for 1 h and then stimulated with LPS (5 μg/mL) for 4 h. Human microglial cells (HMC3) after pretreatment with test compounds for 1 h were stimulated with LPS (10 μg/mL)/TNFα (50 ng/mL) for 24 h. In both microglial models, when the CB2R antagonist (SR144528, 1 μM) was administered, it was added 15 min before agonist administration.

#### Pharmacological *In Vivo* Study

Male CD-1 albino mice (Envigo, Varese, Italy) weighing approximately 22–25 g at the beginning of the experimental procedure were used. Animals were housed in CeSAL (Centro Stabulazione Animali da Laboratorio, University of Florence) and used at least 1 week after their arrival. 10 mice were housed per cage (size 26 × 41 cm); animals were fed a standard laboratory diet and tap water ad libitum and kept at 23 ± 1 °C with a 12 h light/dark cycle, light at 7 a.m. All animal manipulations were carried out according to the Directive 2010/63/EU of the European Parliament and of the European Union Council (22 September 2010) on the protection of animals used for scientific purposes. The ethical policy of the University of Florence complies with the Guide for the Care and Use of Laboratory Animals of the US National Institutes of Health (NIH Publication no 85-23, revised 1996; University of Florence assurance number: A5278-01). Formal approval to conduct the experiments described was obtained from the Animal Subjects Review Board of the University of Florence. Experiments involving animals have been reported according to ARRIVE guidelines.^76^ All efforts were made to minimize animal suffering and to reduce the number of animals used.

Mice treated with oxaliplatin (2.4 mg kg^–1^) were administered *i.p.* on days 1–2, 5–9, 12–14 (10 *i.p*. injections).^35,43,77^ oxaliplatin was dissolved in a 5% glucose solution. Control animals received an equivalent volume of vehicle. Behavioral tests were performed on day 15.

#### Cold Plate Test

Animals were placed in a stainless-steel box (12 cm × 20 cm × 10 cm) with a cold plate as a floor. The temperature of the cold plate was kept constant at 4 ± 1 °C. Pain-related behavior (licking of the hind paw) was observed, and the time (seconds) of the first sign was recorded. The cut-off time of the latency of paw lifting or licking was set at 60 s.

#### Compound Administration

**FD-22a** (1, 5, and 20 mg kg^–1^) was dissolved in 1% carboxymethylcellulose and orally administered. The dose of FD-22a was chosen based on previous studies reporting the antinociceptive activity of EC-21a in a preclinical model of neuropathic pain.^35^ Measurements were performed 15, 30, 45, 60, and 75 min after injection. Control mice were treated with vehicle. The selective CB2R antagonists SR144528 (Tocris Bioscience, UK) and MC21^45^ were dissolved in saline solution with 5% DMSO and 5% Tween 20. Antagonists were administered *i.p.* 15 min before **FD-22a** (20 mg kg^–1^ p.o). The dose of SR144528 was according to previously published articles.^78,79^

#### Statistical Analysis

[^3^H]CP55, 940 radioligand competition binding data are provided as the % change from maximal ^3^H bound (i.e., 100%). Data for HitHunter cAMP and PathHunter βarrestin2 data are shown as the % of the maximal CP55, 940 response (i.e., 100%). Estimates of *K*_i_, EC_50_, *E*_min_, and *E*_max_ were determined using a three-parameter nonlinear regression with the Hill Slope being constrained to 1 (GraphPad, Prism, v. 9.0). In circumstances where *E*_max_ could not be determined because a maximum plateau was not observed with the treatment group, the mean of the maximum observed response was reported as the *E*_max_. Analyses of variance (ANOVA), followed by appropriate posthoc tests, was used for statistical analyses of cAMP, βarrestin2 and radioligand binding as indicated (*p* < 0.05 determined to be significant; Tables S1–S4). Values are presented as the mean ± S.E.M. or 95% C. I., as indicated in tables and figure legends.

The results of additional *in vitro* experiments on microglial cells are expressed as the mean ± standard error of the mean (S.E.M.). Statistical analyses were performed using commercial software (GraphPad Prism, San Diego, CA, USA) using ordinary one-way ANOVA followed by Tukey’s honestly significant difference posthoc test. Differences for which *p* < 0.05 were considered significant.

Behavioral measurements were performed on 16 mice for each treatment carried out in 2 different experimental sets (8 animals for single experimental session). Results were expressed as mean ± S.E.M. The analysis for variance of behavioral data was performed by one-way ANOVA, while Bonferroni’s significant difference procedure was used for posthoc comparison. *p* values of less than 0.05 or 0.01 were considered significant (Table S5). Investigators were blind to all experimental procedures. Data were analyzed using the “Origin 9” software (OriginLab, Northampton, USA).

### Computational Studies

#### Cavities Identification

A site search analysis was performed using FLAP software,^1^ using all CB2R structures available on the PDB website.^80^ 3D complexes 5ZTY,^49^ 6KPC,^50^ 6KPF,^50^ and 6PT0^51^ were checked, and all broken residues were mutated using Maestro^81^ and optimized. For 5ZTY, 6KPC, and 6KPF structures, few missing atoms were localized in extracellular or intracellular disordered regions. In 6PT0, neither missing atoms nor multiple conformations were detected. All CB2 structures were imported into FLAP by applying the predefined FLAP base filters for pdb files. FLAPsite^48^ algorithm was then applied for the identification of protein cavities, using 0,6,1 as the number of additional trials, sensitivity, and erosion values, respectively. This nondefault parameter setting aimed to force detection also of those cavities located on the surface. The cavities were compared within all CB2 structures, taking into account the different activation states.

#### Docking

Crystallographic structures of 5ZTY,^49^ 6KPC,^50^ and 6PT0^52^ relative to inactive, intermediate, and active conformations, already refined through Maestro,^81^ had been used for docking **FM-6b**, **EC-21a**, and all **FD** compounds using GOLD program.^59^ For docking **FM-6b**, the region of interest was defined in such a manner that the protein contained all the residues within 10 Å of the ligand. For docking **EC-21a**, the same procedure was applied to the CB2-ligand orthosteric complex containing the crystallographic ligand in the binding site, searching the possible allosteric cavity for **EC-21a**. All **FD** compounds were subjected to a docking in the empty orthosteric cavity, with a scaffold constraint of strength 5 on the **FM-6b** docked pose, with the aim to simulate the agonist binding of the orthosteric pharmacophoric portion of **FD** compounds and check the consequent disposition of the **EC21a**-derived allosteric tail. In the same condition, the free calculation without any scaffold constraint was also performed. The “allow early termination” command was always deactivated. All ligands were submitted to 40 Genetic Algorithm runs using ChemScore, ASP, PLP, and GoldScore fitness functions, clustering the output orientations on the basis of an RMSD distance of 1.5 Å. The default GOLD parameters were used for all other variables. Docking results were analyzed by using Chimera 1.15.^82^ The distance among the three binding sites was calculated defining pseudoatoms in the ligand centers through Maestro.^81^

## Supporting Information Available

Statistics for inhibition of forskolin-stimulated cAMP and βarrestin2 recruitment, statistics for inhibition of forskolin-stimulated cAMP, statistics for [^3^H]CP55,940 binding Emin, statistics for forskolin-stimulated cAMP, statistics for behavioral tests performed in vivo, summary of receptor cavities, **EC-21a** docking, docking of FD compounds, comparison between the potential CB2R allosteric region with the superimposed CBR1, ^1^H and ^13^C spectra of the final compounds, and HPLC chromatogram of **FD-22a** (PDF)
Molecular Formula Strings (CSV)
Structure of all complex (PDB)

## Supplementary Material

jm2c00582_si_002.csv

jm2c00582_si_003.pdb

## Abbreviations

ECS: endocannabinoid system
CB_1_R: type-1 cannabinoid receptor
CB_2_R: type-2 cannabinoid receptor
ECs: endocannabinoids
AEA: anadamide
2-AG: 2-arachidonoylglycerol
MAGL: monoacylglycerol lipase
MeOH: methanol
SOCl_2_: thionyl chloride
DMF: *N*,*N*-dimethylformamide
DMSO: dimethyl sulfoxide
NEt_3_: triethylamine
CHCl_3_: chloroform
CH_2_Cl_2_: dichloromethane
ACN: acetonitrile
TBTU: 2-(1*H*-benzotriazole-1-yl)-1,1,3,3-tetramethylaminium tetrafluoroborate
DME: dimethoxy ethane
TLC: thin-layer chromatography
4-NPA: 4-nitrophenylacetate
EDTA: ethylenediaminetetraacetic acid
BSA: bovine serum albumin
FBS: fetal bovine serum
