# Supplementary material for: Design, Synthesis, and Biological Activity of New CB2 Receptor Ligands: from Orthosteric and Allosteric Modulators to Dualsteric/Bitopic Ligands
Source: J Med Chem. 2022 Jul 18;65(14):9918–38. doi: 10.1021/acs.jmedchem.2c00582 (PMC10168668; doi:10.1021/acs.jmedchem.2c00582)

Supporting Information

Design, synthesis and biological activity of new CB2 receptor ligands: from orthosteric and allosteric modulators to dualsteric/bitopic ligands

Francesca Gado,[1,#,¥] Rebecca Ferrisi,[1,#] Beatrice Polini,[1,2] Kawthar A. Mohamed,[3] Caterina Ricardi,[2] Elena Lucarini,[4] Sara Carpi,[1,5] Federica Domenichini,[1] Lesley A. Stevenson,[6] Simona Rapposelli,[1,7] Giuseppe Saccomanni,[1] Paola Nieri,[1] Gabriella Ortore,[1] Roger G. Pertwee,[6] Carla Ghelardini,[4] Lorenzo Di Cesare Mannelli,[4] Grazia Chiellini,[2,7] Robert B. Laprairie,[3,8*] Clementina Manera[1,7,*]*

[1]Department of Pharmacy, University of Pisa, 56126 Pisa, Italy;

[2]Department of Pathology, University of Pisa, 56126 Pisa, Italy;

[3]College of Pharmacy and Nutrition, University of Saskatchewan, Saskatoon, SK S7N 5E5, Canada.

[4]Department of Neuroscience, Psychology, Drug Research and Child Health, Section of Pharmacology and Toxicology, University of Florence, 50139 Florence, Italy.

[5]NEST, Istituto Nanoscienze-CNR and Scuola Normale Superiore, Piazza San Silvestro 56126 Pisa, Italy.

[6]Institute of Medical Sciences, University of Aberdeen, AB25 2ZD Aberdeen, Scotland, UK;

[7]CISUP, Centre for Instrumentation Sharing Pisa University, Lungarno Pacinotti 43, 56126 Pisa, Italy;

[8]Department of Pharmacology, College of Medicine, Dalhousie University, Halifax, Nova Scotia, B3H 4R2, Canada.

Corresponding Authors

*Clementina Manera: clementina.manera@unipi.it;

*Robert B. Laprairie: robert.laprairie@usask.ca;

*Grazia Chiellini: grazia.chiellini@unipi.it

CONTENTS OF SUPPORTING INFORMATION

S3 **Extended Data Table S1. Statistics for inhibition of forskolin-stimulated cAMP and βarrestin2 recruitment.**

S4 **Extended Data Table S2. Statistics for inhibition of forskolin-stimulated cAMP.**

S5 **Extended Data Table S3. Statistics for [^{3}H]CP55,940 binding E_{min}.; Extended Data Table S4. Statistics for forskolin-stimulated cAMP.**

S6, S7 **Extended Data Table S5. Statistics for Behavioral Tests performed in vivo.**

S7 **Figure S1**: FM-6b, EC-21a, FD-22a, and FD-24a inhibit cAMP accumulation in CHO cells lacking CB2R

S8 **Figure S2**. Summary of receptor cavities

S8 **Figure S2**. **EC-21a** docking.

S9 **Figure S3**. Docking of FD-compounds

S9 **Figure S4**. Comparison between the potential CB2R allosteric region with the superimposed CBR1

S10-S18 ^{1}H- and ^{13}C-NMR Spectra of compounds **FD-22a, FD-24a, FD-25a, FD-30a FD-27a FD-28a, FD-32a** and **FD-31a** and HPLC chromatogram of **FD-22a**

Extended Data Table S1. Statistics for inhibition of forskolin-stimulated cAMP and βarrestin2 recruitment.

	ANOVA	F(DFn,DFd)	p value
**Figure 2**	ME Drug	F(7,80)=113.0	< 0.0001
	ME Assay	F(1,80)=3.248	< 0.0001
	INT	F(7,80)=17.39	< 0.0001
**Inhibition of FSK-stimulated cAMP (% CP55,940) E_{max}**			
**Figure 2a,b**	**Comparison**	**Adjusted p value**	
	CP55,940 vs. EC-21a	< 0.0001	
	CP55,940 vs. FM-6b	0.0001	
	CP55,940 vs. FD-22a	< 0.0001	
	CP55,940 vs. FD-24a	< 0.0001	
	CP55,940 vs. FD-25a	< 0.0001	
	CP55,940 vs. FD-27a	< 0.0001	
	CP55,940 vs. FD-32a	< 0.0001	
**βarrestin2 recruitment (% CP55,940) E_{max}**			
**Figure 2c,d**	**Comparison**	**Adjusted p value**	
	CP55,940 vs. EC-21a	< 0.0001	
	CP55,940 vs. FM-6b	< 0.0001	
	CP55,940 vs. FD-22a	< 0.0001	
	CP55,940 vs. FD-24a	< 0.0001	
	CP55,940 vs. FD-25a	< 0.0001	
	CP55,940 vs. FD-27a	< 0.0001	
	CP55,940 vs. FD-32a	< 0.0001	

CB2R activity was quantified for cAMP inhibition using the DiscoveRx HitHunter assay (CHO *h*CB2R) in cells treated with compounds for 90 min, and for βarrestin2 recruitment using the DiscoveRx PathHunter assay (CHO *h*CB2R) in cells treated with compounds for 90 min. Data were fit to a variable slope (three-parameter) non-linear regression in GraphPad (v. 9). Data for EC_{50} were analyzed by mean with 95% confidence interval (C.I.) and assessed by non-overlapping 95% C.I. (Table 1, no further analysis here). Data for E_{max} were analyzed by mean ± S.E.M. with two-way ANOVA followed by Dunnett's post-hoc test (within assay). n = 6 independent experiments performed in triplicate. Data from this Table are graphed in Figure 2 and presented in Table 1.

Extended Data Table S2. Statistics for inhibition of forskolin-stimulated cAMP.

	ANOVA	F(DFn,DFd)	p value
**Figure 3**	ME Treatment	F(9,35)=19.94	< 0.0001
**Inhibition of FSK-stimulated cAMP (% CP55,940) E_{max}**			
**Figure 3**	**Comparison**	**Adjusted p value**	
	CP55,940 vs. FM-6b	0.209	
	CP55,940 vs. EC-21a	< 0.0001	
	CP55,940 vs. 10 nM FM-6b + EC-21a	0.0087	
	CP55,940 vs. FD-22a	0.0002	
	CP55,940 vs. 50 nM FD-22a + EC-21a	< 0.0001	
	CP55,940 vs. 100 nM SR144528 + FD-22a	< 0.0001	
	CP55,940 vs. FD-24a	< 0.0001	
	CP55,940 vs. 5 nM FD-24a + EC-21a	0.9808	
	CP55,940 vs. 100 nM SR144528 + FD-24a	< 0.0001	
	FM-6b vs. EC-21a	< 0.0001	
	FM-6b vs. 10 nM FM-6b + EC-21a	0.7043	
	FM-6b vs. FD-22a	0.209	
	FM-6b vs. 50 nM FD-22a + EC-21a	0.0004	
	FM-6b vs. 100 nM SR144528 + FD-22a	0.0459	
	FM-6b vs. FD-24a	0.0046	
	FM-6b vs. 5 nM FD-24a + EC-21a	0.9808	
	FM-6b vs. 100 nM SR144528 + FD-24a	0.0041	
	EC-21a vs. 10 nM FM-6b + EC-21a	0.0007	
	EC-21a vs. FD-22a	0.0001	
	EC-21a vs. 50 nM FD-22a + EC-21a	0.8329	
	EC-21a vs. 100 nM SR144528 + FD-22a	0.0512	
	EC-21a vs. FD-24a	0.01	
	EC-21a vs. 5 nM FD-24a + EC-21a	< 0.0001	
	EC-21a vs. 100 nM SR144528 + FD-24a	0.3329	

CB2R activity was quantified for cAMP inhibition using the DiscoveRx HitHunter assay (CHO *h*CB2R) in cells treated with compounds for 90 min. Data were fit to a variable slope (three-parameter) non-linear regression in GraphPad (v. 9). Data for EC_{50} were analyzed by mean with 95% confidence interval (C.I.) and assessed by non-overlapping 95% C.I. (Table 2, no further analysis here). Data for E_{max} were analyzed by mean ± S.E.M. with one-way ANOVA followed by Tukey's post-hoc test. n = 3-6 independent experiments performed in triplicate. Data from this Table are graphed in Figure 3 and presented in Table 2.

Extended Data Table S3. Statistics for [^{3}H]CP55,940 binding E_{min}.

	ANOVA	F(DFn,DFd)	p value
**Figure 4**	ME Drug	F(4,20)=128.8	< 0.0001
	ME Receptor	F(1,20)=20.14	0.0002
	INT	F(4,20)=15.05	< 0.0001
**[^{3}H]CP55,940 binding at hCB1R (% [^{3}H]CP55,940 bound) E_{min}**			
**Figure 4A**	**Comparison**	**Adjusted p value**	
	CP55,940 vs. EC-21a	< 0.0001	
	CP55,940 vs. FM-6b	0.0064	
	CP55,940 vs. FD-22a	< 0.0001	
	CP55,940 vs. FD-24a	< 0.0001	
**[^{3}H]CP55,940 binding at hCB2R (% [^{3}H]CP55,940 bound) E_{min}**			
**Figure 4B**	**Comparison**	**Adjusted p value**	
	CP55,940 vs. EC-21a	< 0.0001	
	CP55,940 vs. FM-6b	0.3338	
	CP55,940 vs. FD-22a	0.0003	
	CP55,940 vs. FD-24a	< 0.0001	

[^{3}H]CP55,940 binding to CB1R and CB2R from CHO-K1 cells were quantified as described in Figure 4 and Table 3. Data were fit to a variable slope (three-parameter) non-linear regression in GraphPad (v. 9). Data for K_i were analyzed by mean with 95% confidence interval (C.I.) and assessed by non-overlapping 95% C.I. (Table 3, no further analysis here). Data for E_{min} were analyzed by mean ± S.E.M. with two-way ANOVA followed by Dunnett's post-hoc test (within assay). n = 3 independent experiments performed in duplicate. Data from this Table are graphed in Figure 4 and presented in Table 3.

Extended Data Table S4. Statistics for forskolin-stimulated cAMP.

	ANOVA	F(DFn,DFd)	p value
**Figure 4**	ME Treatment	F(6,21)=434.2	< 0.0001
	Residual	21	
**[^{3}H]CP55,940 binding at hCB1R (% [^{3}H]CP55,940 bound) E_{min}**			
**Figure 4A**	**Comparison**	**Adjusted p value**	
	+10 μM FSK vs. Untreated	< 0.0001	
	+10 μM FSK vs. CP55,940	0.9593	
	+10 μM FSK vs. FM-6b	< 0.0001	
	CP55,940 vs. FD-22a	< 0.0001	
	CP55,940 vs. FD-24a	< 0.0001	

cAMP accumulation was quantified using the DiscoveRx HitHunter assay (CHO-K1 cells) in cells treated with compounds for 90 min. Data were analyzed in GraphPad (v. 9). Data are mean ± S.E.M. with one-way ANOVA followed by Dunnett's post-hoc test. n = 4 independent experiments performed in triplicate. Data from this Table are graphed in Figure S1.

Extended Data Table S5. Statistics for Behavioral Tests performed in vivo.

	Experimental groups comparison	Time (min)	p value
**Fig. 9**	vehicle + vehicle vs oxaliplatin + vehicle	0	0.00134
		15	< 0.0001
		30	< 0.0001
		45	< 0.0001
		60	< 0.0001
		75	< 0.0001
	oxaliplatin + vehicle vs oxaliplatin + FD22a 1 mg kg^{-1}	0	0.83272
		15	0.80277
		30	0.2073
		45	0.3597
		60	0.22678
		75	0.73019
	oxaliplatin + vehicle vs oxaliplatin + FD22a 5 mg kg^{-1}	0	0.44584
		15	0.03184
		30	0.00116
		45	0.00193
		60	0.1525
		75	0.80889
	oxaliplatin + vehicle vs oxaliplatin + FD22a 20 mg kg^{-1}	0	0.88519
		15	0.04099
		30	0.00462
		45	0.00278
		60	0.02878
		75	0.70909
**Fig. 10**	vehicle + vehicle vs oxaliplatin + FD22a 20 mg kg^{-1} + MC21a 10 mg kg^{-1}	0	< 0.0001
		15	0.00078
		30	< 0.0001
		45	< 0.0001
		60	0.00154
		75	< 0.0001
	vehicle + vehicle vs oxaliplatin + FD22a 20 mg kg^{-1} + SR144428 10 mg kg^{-1}	0	0.00534
		15	< 0.0001
		30	0.00138

45	< 0.0001
60	< 0.0001
75	< 0.0001

Data were analyzed by mean ± S.E.M. with one-way ANOVA followed by Bonferroni's posthoc test. Each value represents the mean of 16 mice per group, performed in 2 different experimental sets. The table is graphed in Figures 9 and 10.

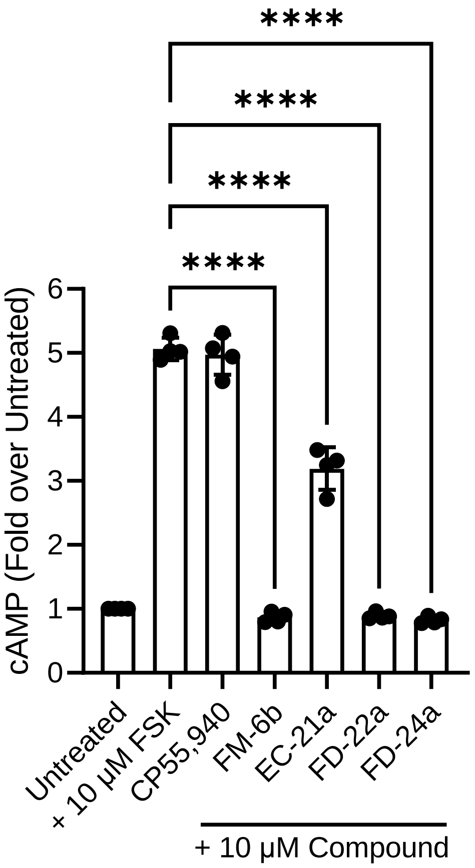

Figure S1: FM-6b, EC-21a, FD-22a, and FD-24a inhibit cAMP accumulation in CHO cells lacking CB2R. Inhibition of FSK-stimulated cAMP accumulation in CHO-K1 cells lacking CB2R. cAMP accumulation data are expressed as fold over untreated cells. Cells were treated with 10 µM FSK and 10 µM compounds simultaneously as indicated. Data are mean ± S.E.M. of 4 independent experiments performed in triplicate. Statistical data for these graphs are presented in Table S4.

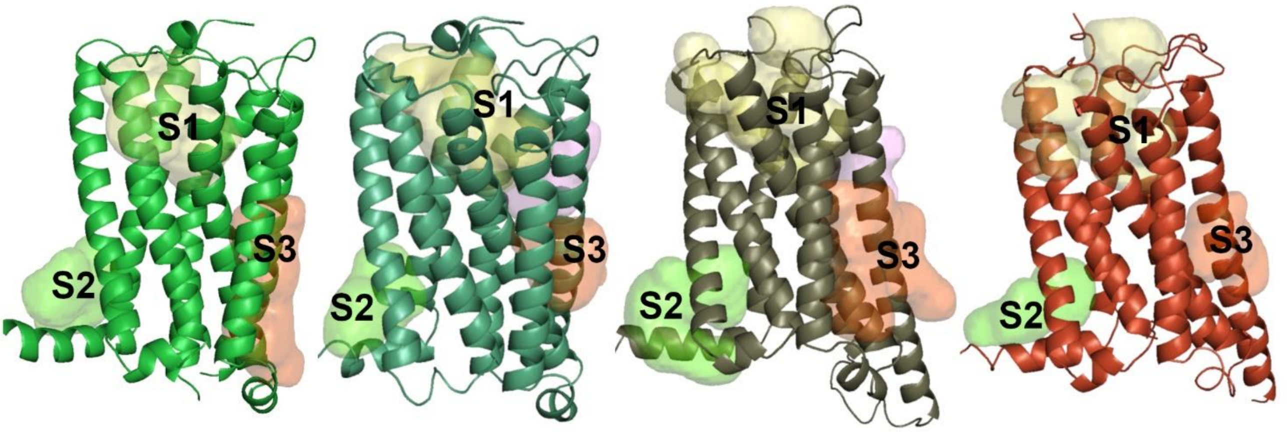

Figure S2: Summary of receptor cavities predicted by Flap program in 5ZTY structure (light green, inactive form), 6KPC (dark green, agonist-bound form), 6PT0 (grey, agonist and Gi-bound complex) and 6KPF (brown, agonist and Gi-bound complex).

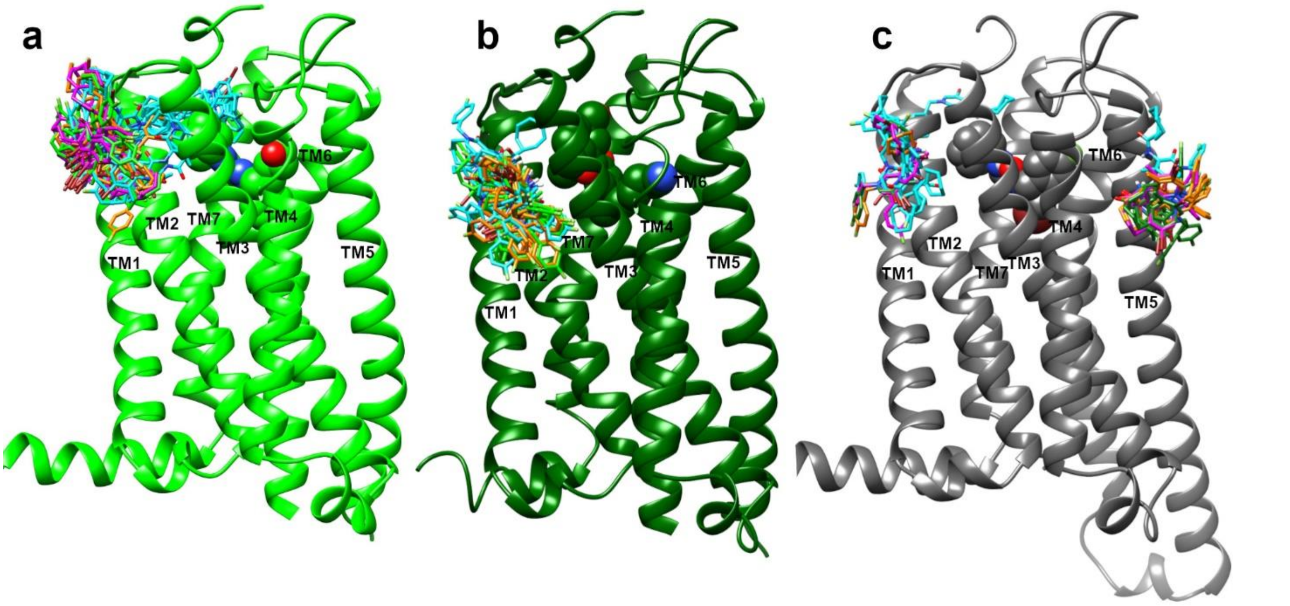

Figure S3: Results of **EC-21a** docking in 5ZTY (a), 6KPC (b) and 6PT0 (c) structures calculated using all GOLD scoring functions: ASP (orange colored), GOLDSCORE (cyan colored), CHEMSCORE (green colored) and PLP (magenta colored)

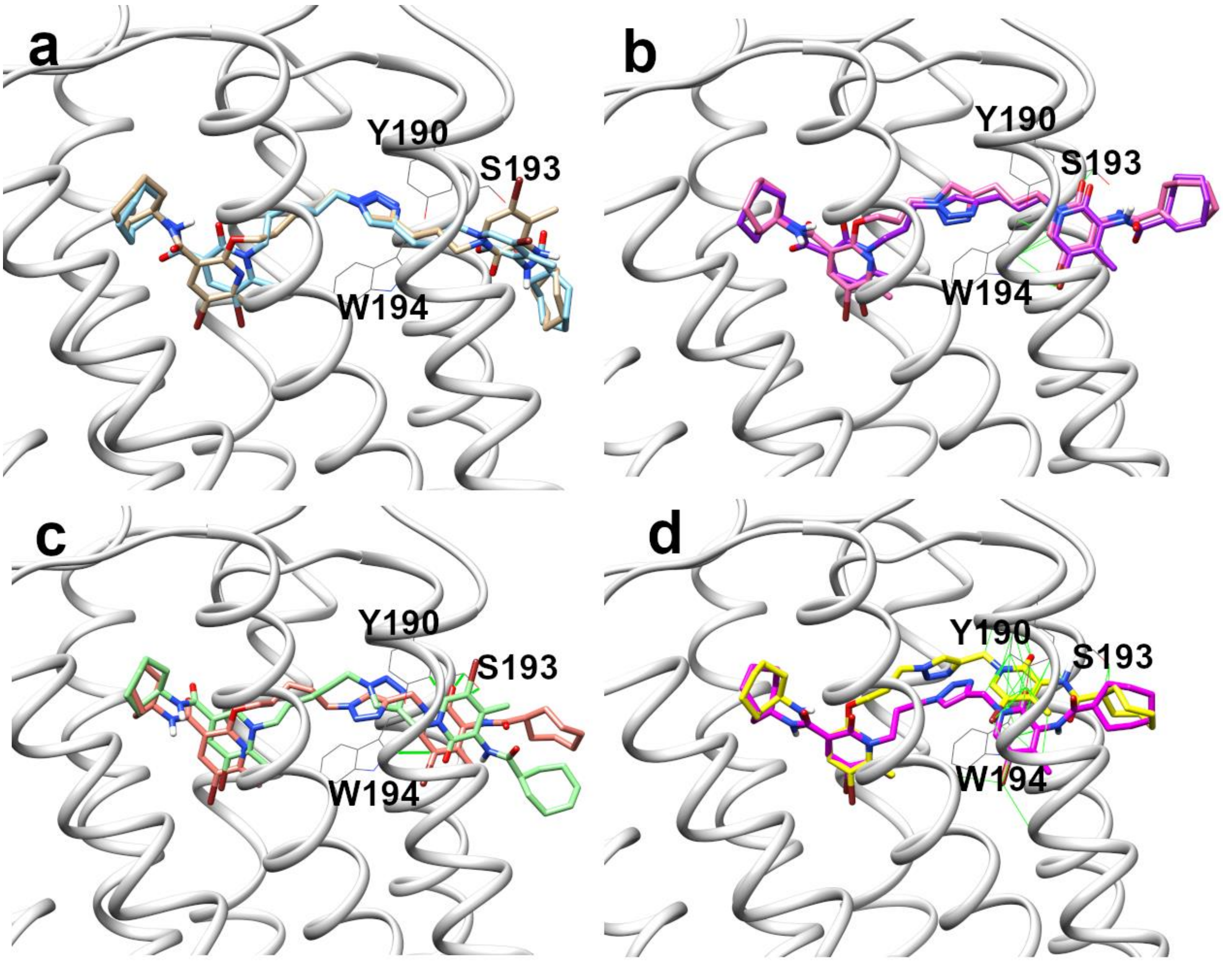

Figure S4. Docking of a) **FD-22a** (light cyan) and **FD-24a** (beige); b) **FD-25a** (purple) and **FD-30a** (pink); c) **FD-27a** (light green) and **FD-28a** (salmon); d) **FD-31a** (yellow) and **FD-32a** (magenta) in 6PT0 structure. Clashes are reported as green lines.

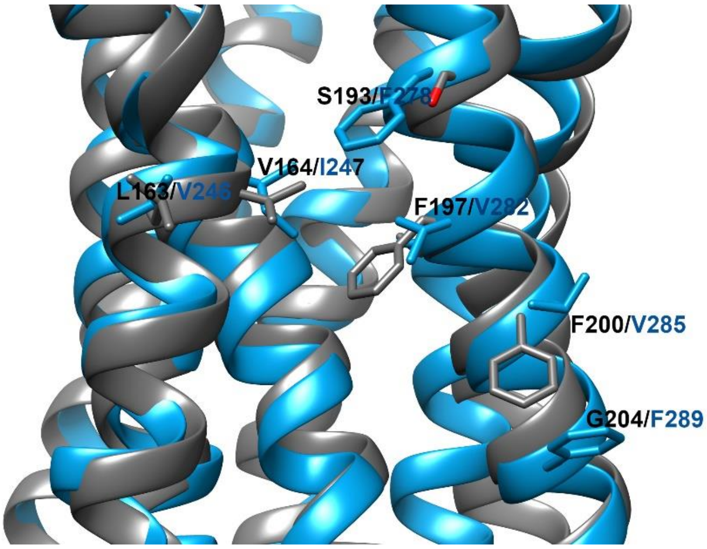

Figure S5. Comparison between the potential allosteric region of CB2R (grey colored) with the superimposed CBR1 (blue colored): non conserved residues are highlighted with analogues colors.

^{1}H-, ^{13}C-NMR Spectra of compounds **FD-22a, FD-24a, FD-25a, FD-30a FD-27a FD-28a, FD-32a** and **FD-31a** and HPLC chromatogram of **FD-22a**

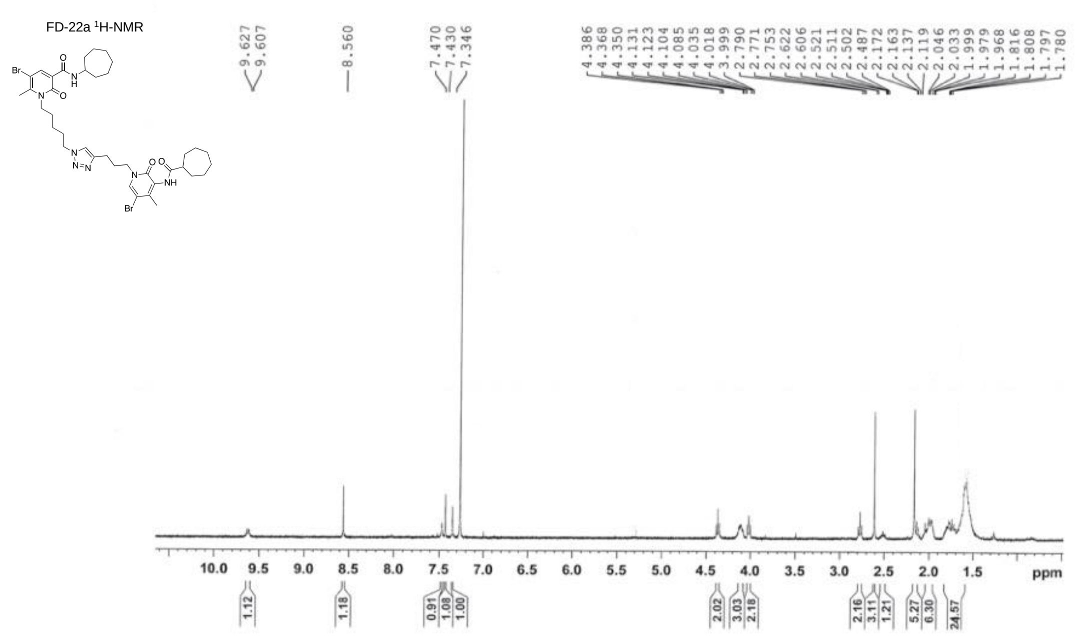

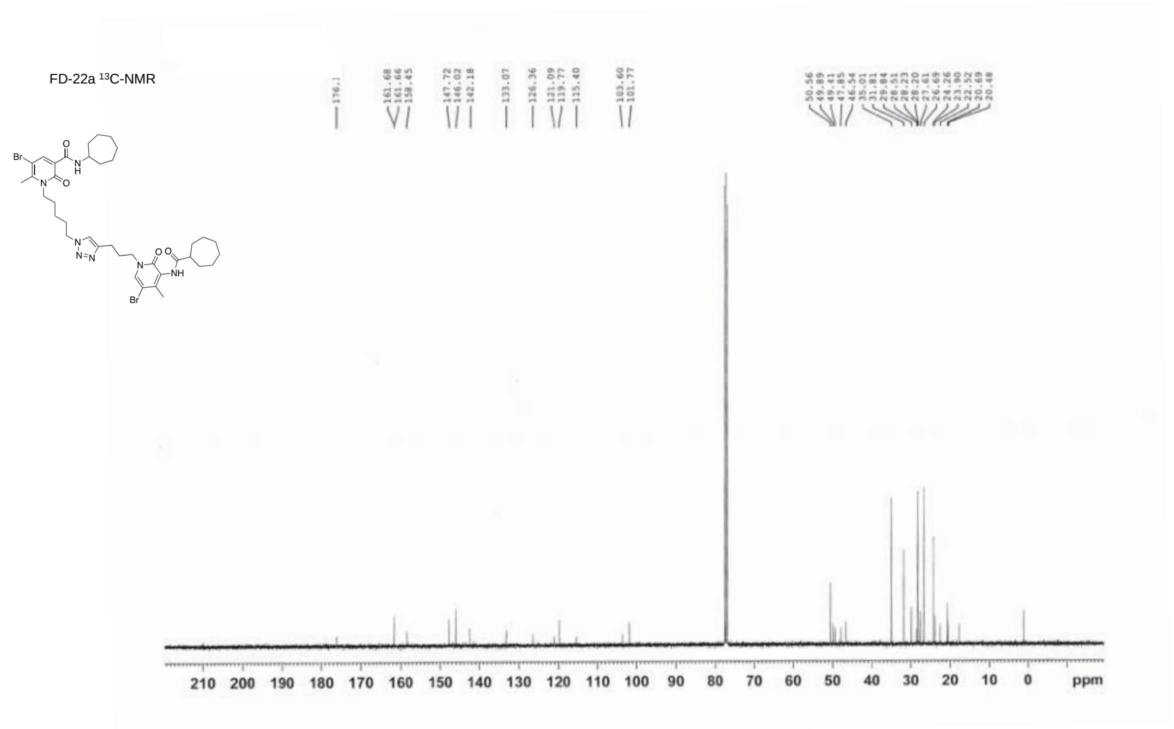

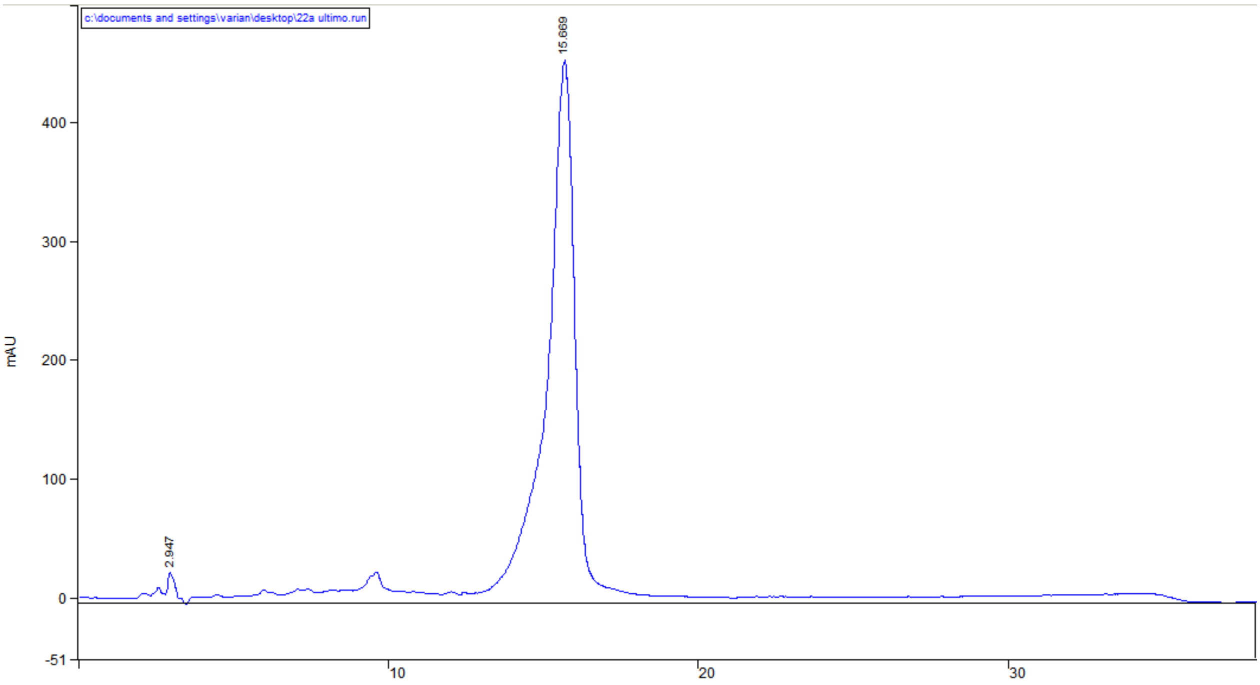

Operator :
Workstation: HPLC
Instrument : Varian Star #1
Channel : 1 = 220.00 nm

Detector Type: 330 UV-Vis. PDA
Bus Address : 71
Sample Rate : 0.63 Hz
Run Time : 51.013 min

** LC Workstation Version 6.41 ** 01938-61c0-ea4-04b0 **

Run Mode : Analysis
Peak Measurement: Peak Area
Calculation Type: Percent

Peak No.	Peak Name	Result ()	Ret. Time (min)	Time Offset (min)	Area (counts)	Sep. Code	Width 1/2 (sec)	Status Codes
1		1.1598	2.947	0.000	1630791	BB	13.3	
2		98.8402	15.669	0.000	138984480	BB	46.9	
	Totals:	100.0000		0.000	140615271			

FD-24a ^{1}H-NMR

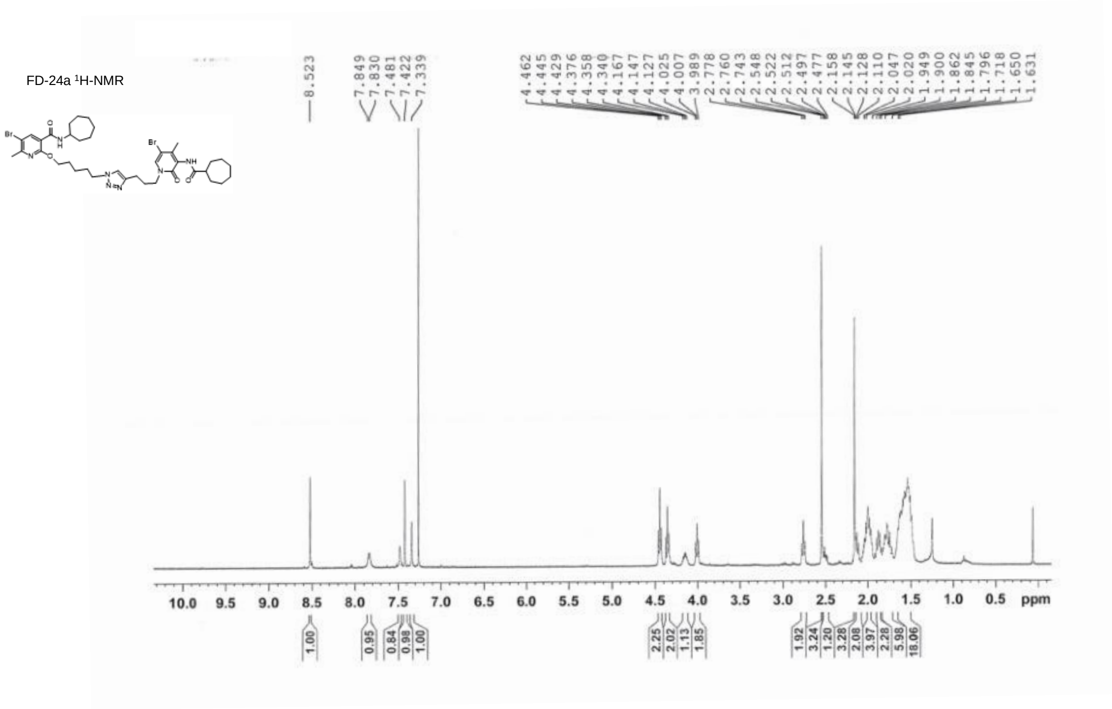

FD-24a ^{13}C-NMR

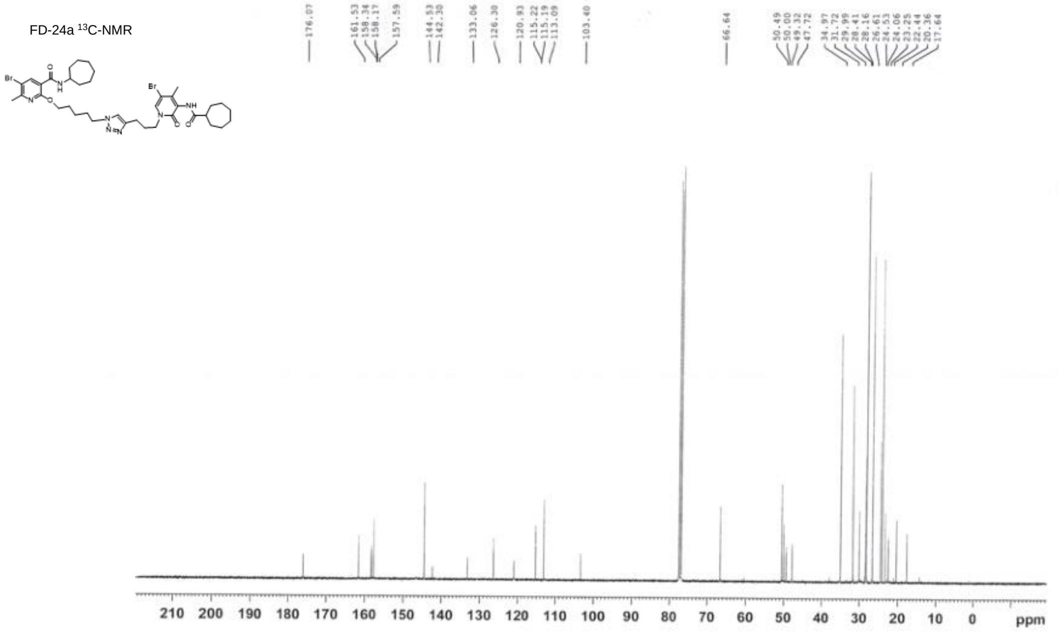

FD-25a ¹H-NMR

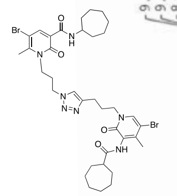

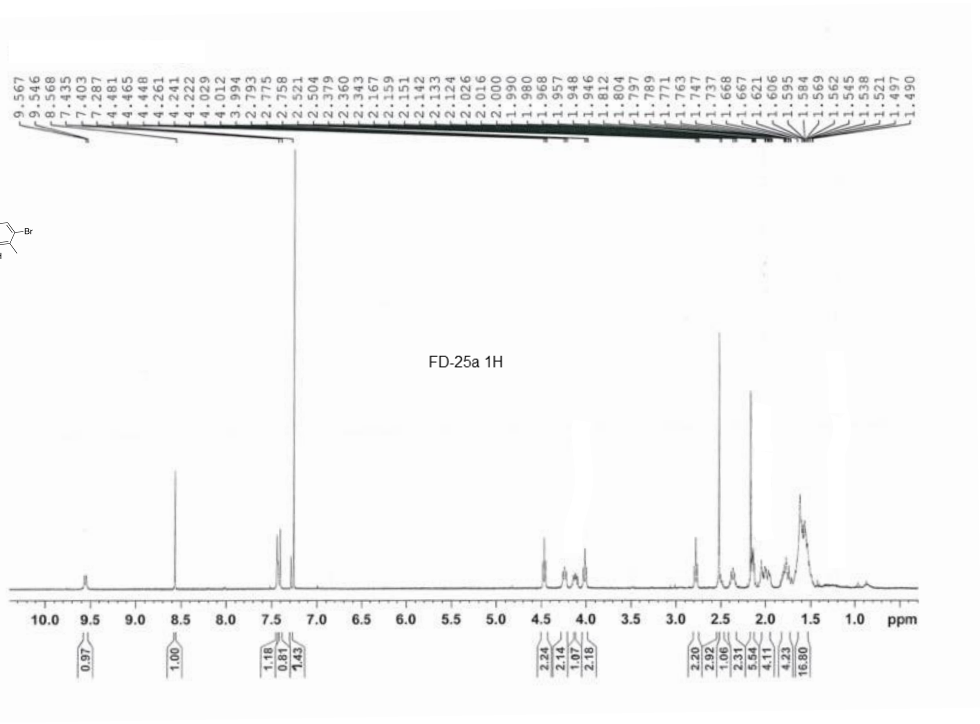

FD-25a ¹³C-NMR

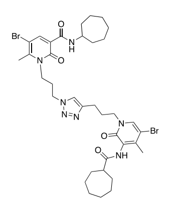

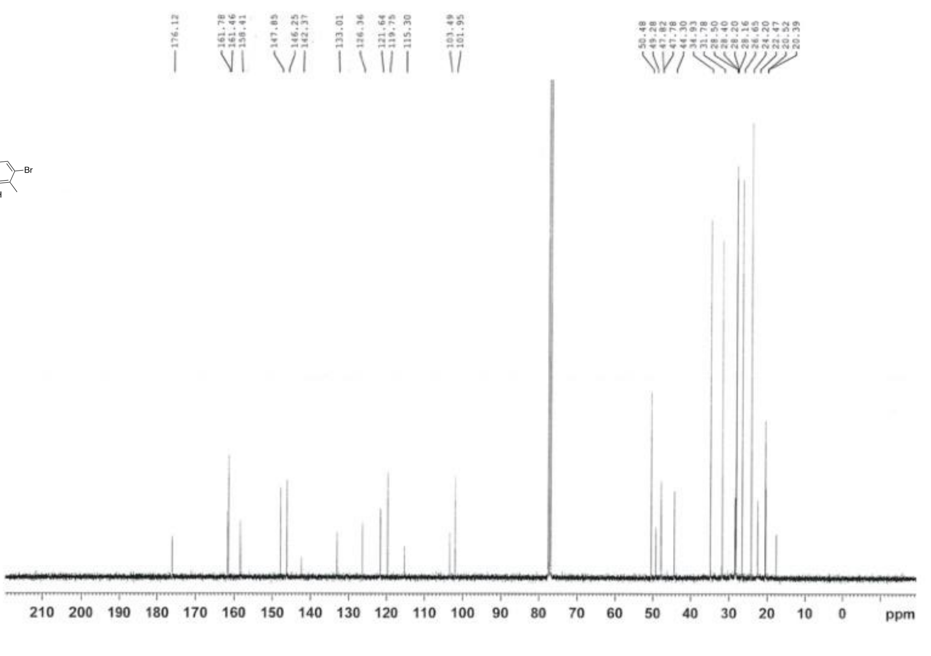

FD-30a ¹H-NMR

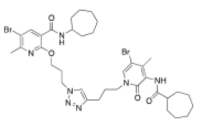

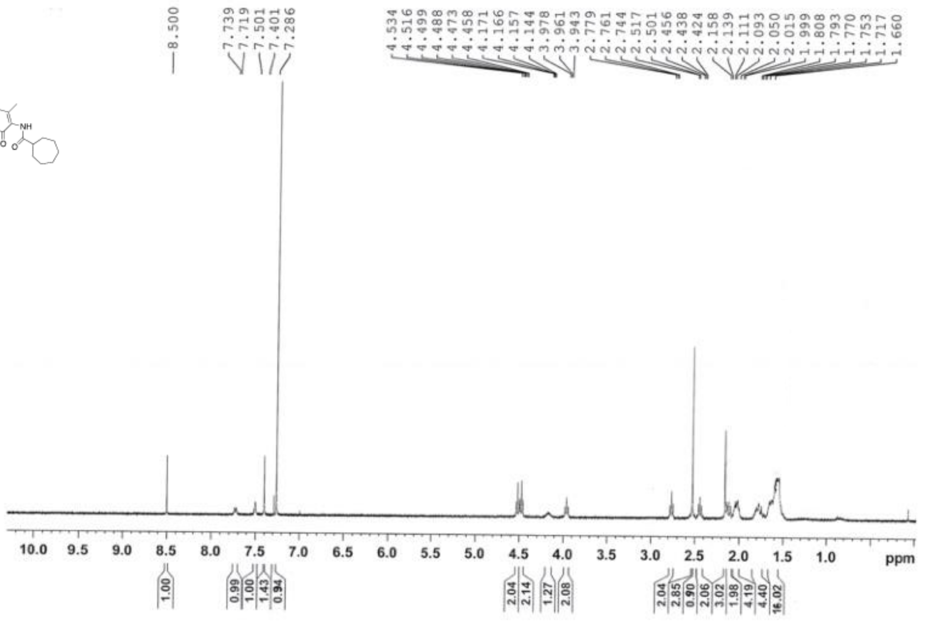

FD-30a ¹³C-NMR

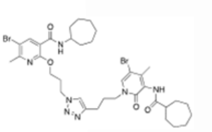

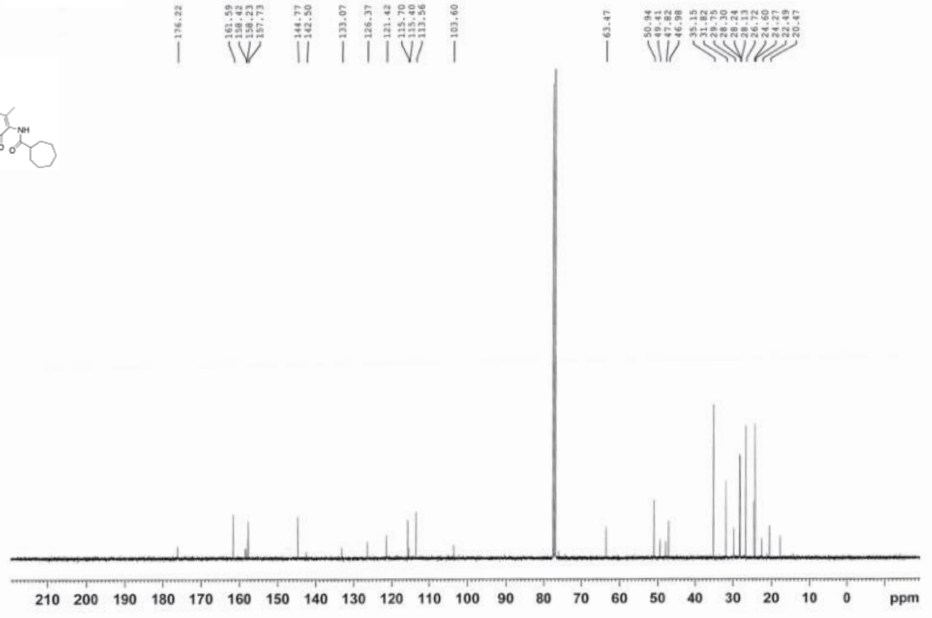

FD-27a ¹H-NMR

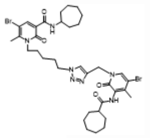

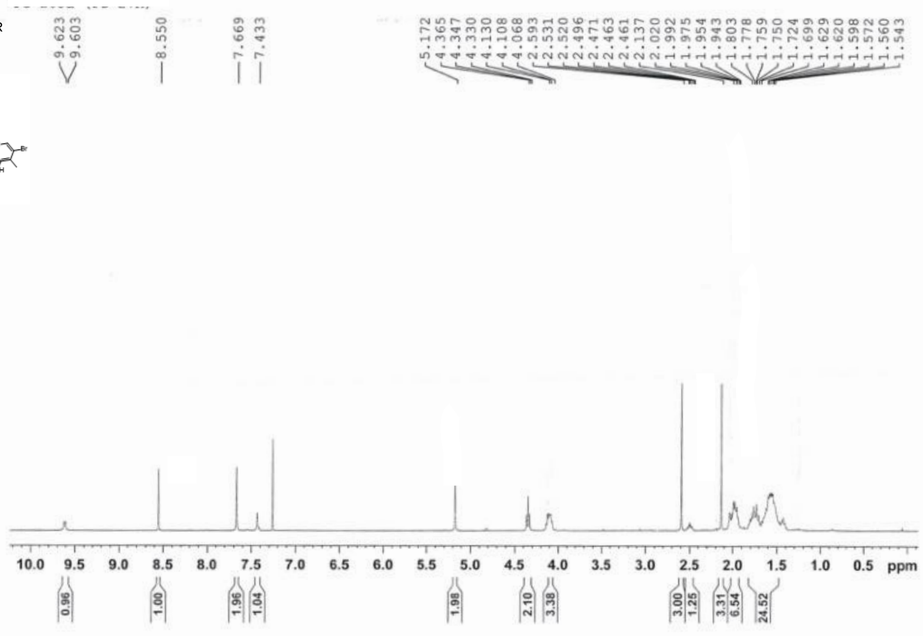

FD-27a ¹³C-NMR

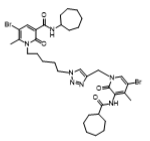

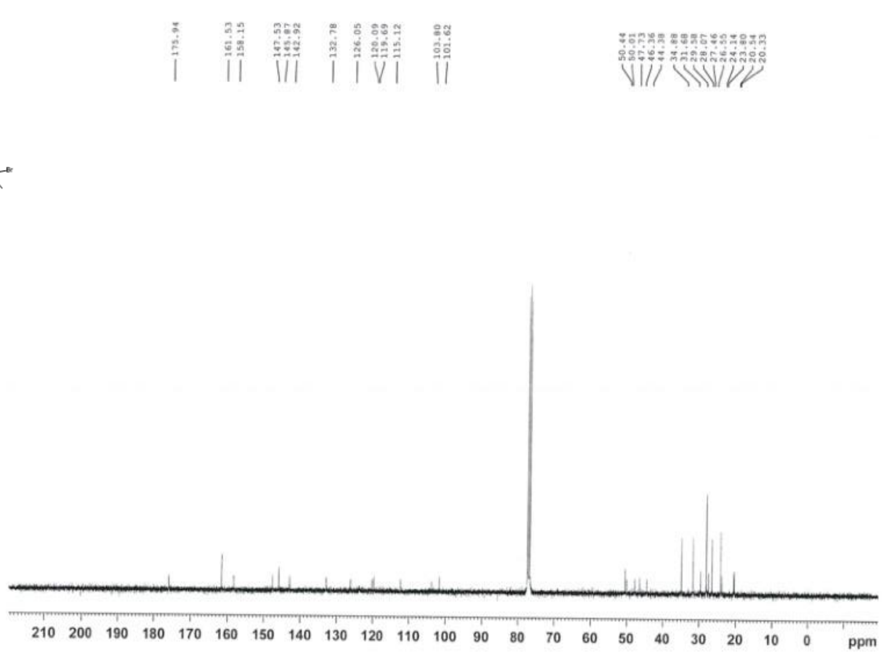

FD-28a ¹H-NMR

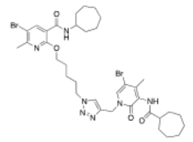

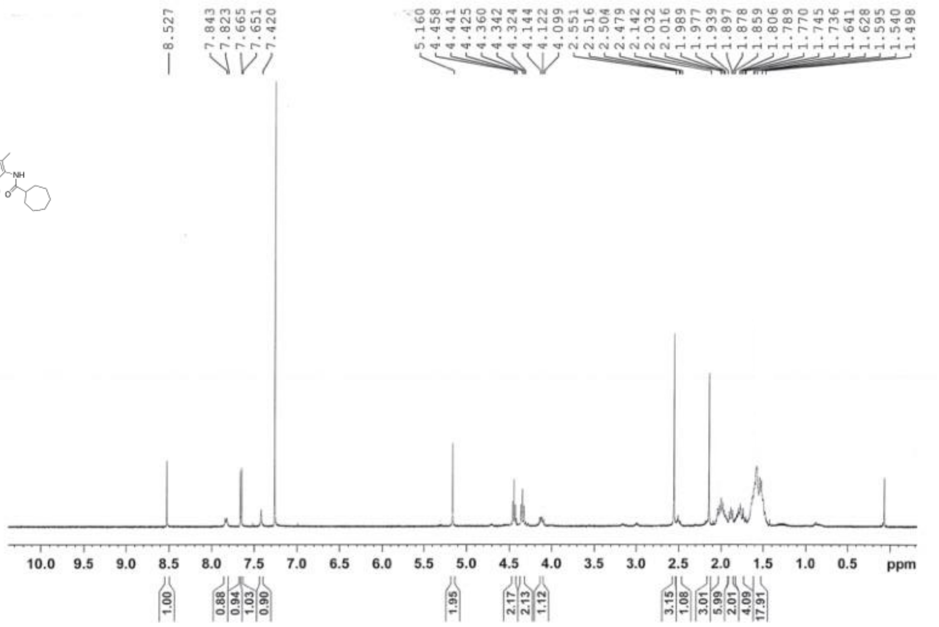

FD-28a ¹³C-NMR

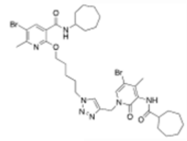

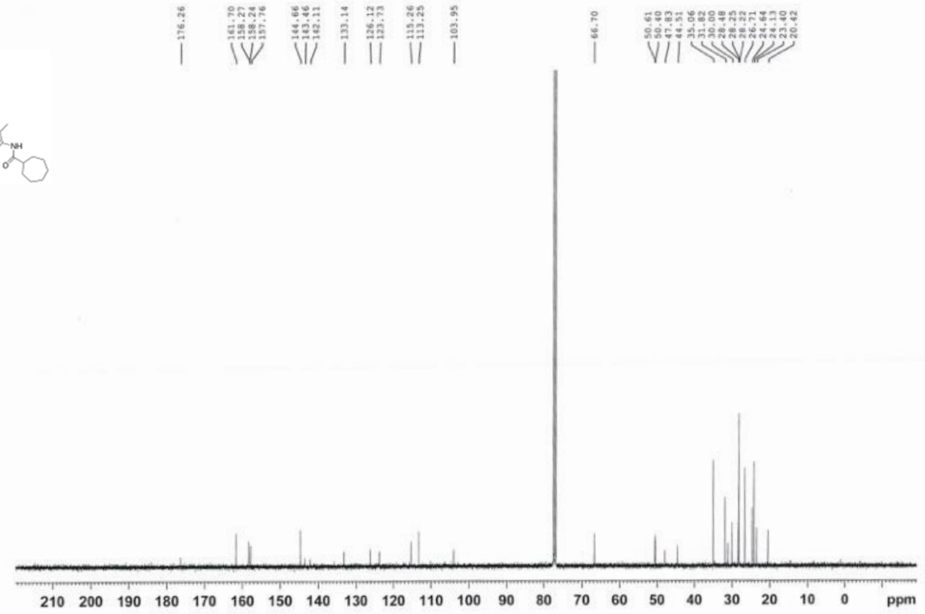

FD-32a ^{1}H-NMR

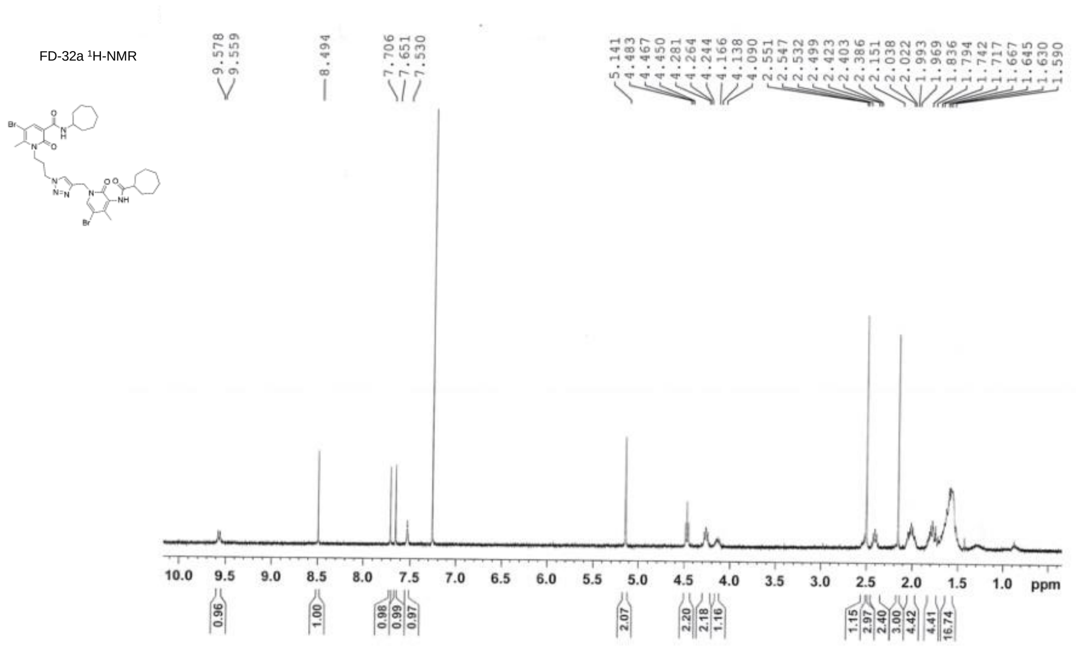

FD-32a ^{13}C-NMR

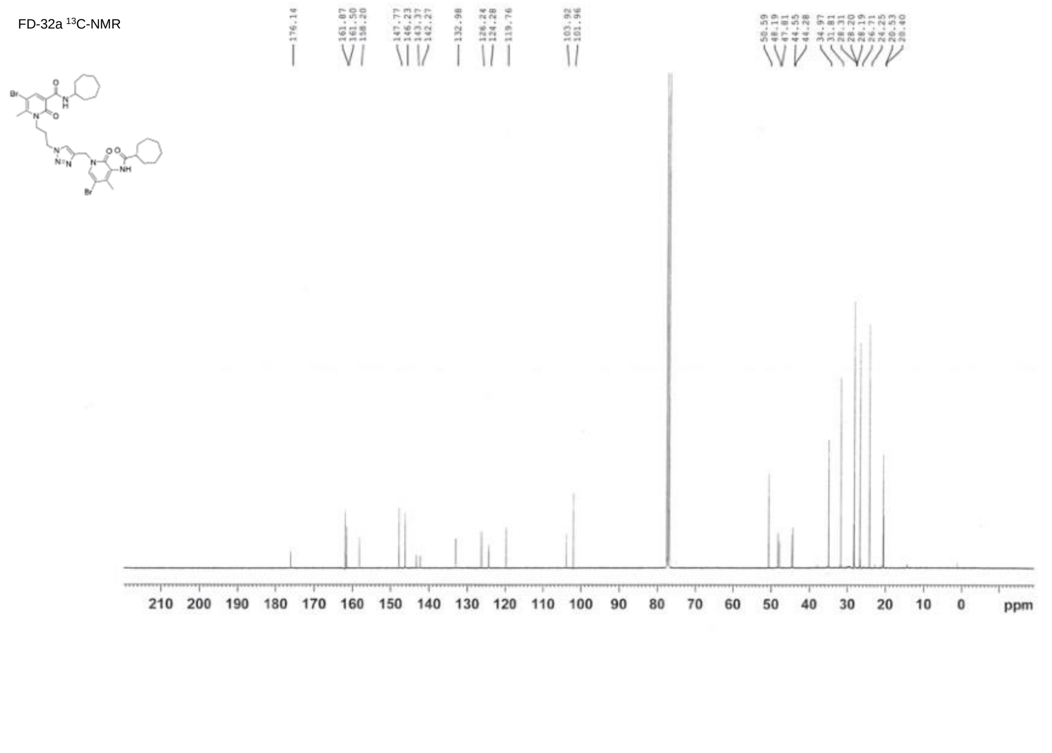

FD-31a ^{1}H-NMR

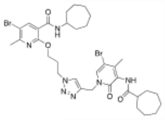

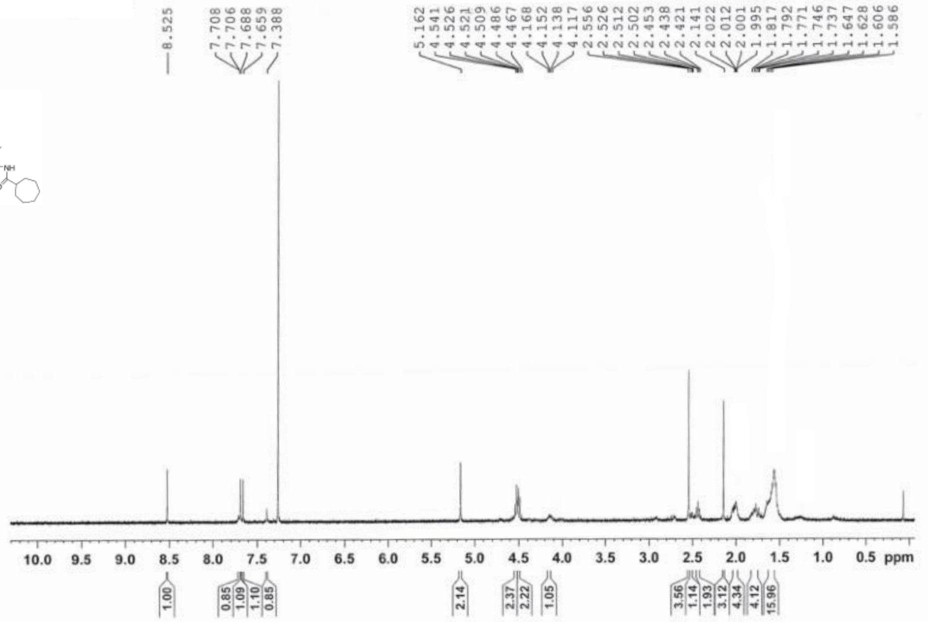

FD-31a ^{13}C-NMR

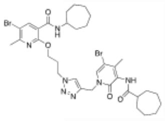

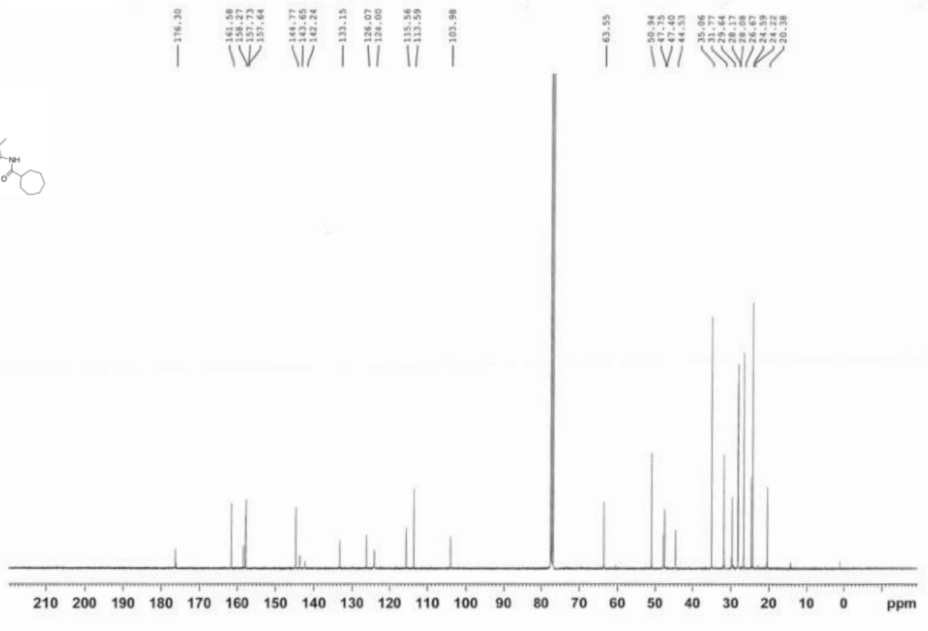

Supplement: Supplementary file 1 — jm2c00582_si_001.pdf [file jm2c00582_si_001.pdf]
